# Genetic variation and genome-enabled selection of white lupin for key seed quality traits

**DOI:** 10.1186/s12864-025-12048-0

**Published:** 2025-10-15

**Authors:** Paolo Annicchiarico, Claudia Osorio, Nelson Nazzicari, Barbara Ferrari, Stefania Barzaghi, Elisa Biazzi, Aldo Tava, Luciano Pecetti, Tommaso Notario, Massimo Romani, Margherita Crosta

**Affiliations:** 1 Research Center for Animal Production and Aquaculture, Council for Agricultural Research and Economics (CREA), viale Piacenza 29, Lodi, 26900 Italy; 2 Instituto de Investigaciones Agropecuarias (INIA), Centro Regional Carillanca, Camino Cajón - Vilcún Km 10, Temuco, Chile

**Keywords:** Genetic variation, Genotype × environment interaction, Protein content, Genetic architecture, Genomic selection, GWAS, *Lupinus albus*, Oil content, Quinolizidine alkaloid, Seed size

## Abstract

**Background:**

White lupin (*Lupinus albus* L.) requires selection for low quinolizidine alkaloid (QA) content and other seed quality traits to become an important high-protein crop. There is limited information on trait variation, genetic architecture, genotype × environment interaction (GEI), relevant genomic areas, and opportunities for genomic selection (GS).

**Results:**

A reference population of sweet-seed breeding lines possessing the *pauper* locus underwent multi-year evaluation for seed weight, protein content and oil content in two regions (Italy and Chile) and evaluation for content of 13 QAs by a gas chromatography-mass spectrometry method in Italy. A second population including landrace genotypes of worldwide origin was evaluated for protein and oil content and seed weight in Italy. We found substantial genetic variation for all traits. Only 24% of the breeding lines displayed total QA content below 200 mg/kg. Lupanine was the main QA, followed by 13α-hydroxylupanine and 13α-angeloyloxylupanine. GEI across regions was large for protein content, moderate for oil content, and low for seed weight, while being always low across cropping years within region. Genotyping-by-sequencing provided 33,473 SNPs for breeding lines and 41,116 SNPs for landrace genotypes. A genome-wide association study highlighted the polygenic control of total QA content and other traits, identified candidate genes and, particularly for protein content, showed inconsistency for significant SNPs across regions or reference populations. Landrace genotypes exhibited weak population structure partly related to phenology and geographic origin. Our results indicated that region-specific selection for seed weight, protein content and oil content is favoured by high broad-sense heritability, high consistency between parent and progeny values, low GEI, absence of high inverse correlations between traits, and high to moderately high intra-population GS predictive ability (0.41–0.80). The application of GS models defined in one region for selection in the other region, or that of GS models trained on the genetically broader landrace population for selection of breeding lines, proved convenient for seed weight, possible with limitations for oil content, and inconvenient for protein content. High predictive ability (0.66) emerged for total QA content.

**Conclusions:**

Our results highlighted opportunities and limits for phenotypic and genome-enabled selection that can help define efficient breeding strategies.

**Supplementary Information:**

The online version contains supplementary material available at 10.1186/s12864-025-12048-0.

## Background

Greater grain legume cultivation is a priority for agriculture in Europe and other regions to enhance its environmental sustainability [1, 2], its self-sufficiency for high-protein feedstuff [3], and its ability to meet the raising demand for healthy and nutritious plant-based foods [4]. White lupin (*Lupinus albus* L.) is a crop with a long history of domestication [5] that has high potential interest in this context, because of its seed crude protein content in the range of 33–47% and several useful nutritional and technological characteristics [6–8]. Its exploitation as a component of functional, healthy or vegan food is reinforced by the positive effects on human health that it can exert with respect to diabetes and glycaemia, hypertension, cardiovascular diseases, and obesity [9, 10]. In addition, the white lupin seed contains 6–13% of oil [7] with excellent food quality due to high levels of unsaturated fatty acids [11, 12]. White lupin has large potential use also as a high-protein feed for ruminants and poultry [13, 14] and in aquaculture [15]. The remarkable agronomic interest of white lupin as a high-protein crop is reinforced by its higher protein yield per unit area compared with other cool-season grain legumes in Southern Europe [16] and other regions [17].

Despite its several positive characteristics, white lupin requires genetic improvement for yield and seed quality characteristics to fully realize its potential importance and increase the economic sustainability of its cultivation [6, 7]. High protein content, which is one of its main assets, ought to be maximized [14] (whereas the selection for specific amino acids is hindered by limited genetic variation [18]). Industrial oil extraction occurs for material with at least 17–18% lipid content, such as soybean seed and rice bran [19], and novel cultivars achieving this content could turn white lupin into a dual-purpose crop (for protein and high-quality food oil) [14]. A large seed (say, >0.50 g), which is mainly needed for its traditional consumption as a snack, is hardly found in modern cultivars while being present in some landraces [20]. These traits are expected to be under polygenic control, as confirmed for protein content by a recent study [21], but the extent of their genetic complexity is largely unknown.

Probably the main and most challenging seed quality trait for white lupin breeders is represented by reduced content of quinolizidine alkaloids (QAs). QAs are a set of secondary metabolites synthesized by lupin species and other plants as a defence mechanism against pathogens and herbivores [22–24]. In addition, QAs in the seed may act as a nitrogen storage means for use by the seedling after germination [25]. QAs confer a bitter taste to lupin seeds and can be toxic for humans and animals [26, 27]. Although their actual level of toxicity for humans is still controversial [27], a threshold of 200 mg/kg of total QA content in lupin-based food products has been fixed by the Health Authorities of various countries [28, 29], whereas a threshold of 500 mg/kg is recommended for animal feed [30]. The former threshold was actually exceeded by some food products upon verification [27, 31, 32], whereas total QA content greater than 500 mg/kg may occur frequently in elite breeding lines [33] and occasionally in commercial cultivars [34–36]. Landrace germplasm displays much higher content of total QAs, albeit with large genetic variation [36–38]. White lupin selection for low content of QAs has essentially relied on the exploitation of the *pauper* locus located on chromosome 18 [14]. A recessive gene encoding for an acyltransferase involved in the early QA pathway has been identified in this locus, associated with a single nucleotide polymorphism (SNP) [39]. This locus has a strong depressive effect on total QA content but does not necessarily reduce its level below 500 mg/kg, owing to different allelic forms [40], non-allelic gene interactions [41], and minor genes that generate a complex trait inheritance pattern mirroring the complexity of the QA biosynthetic pathway [26, 42]. The ability to select efficiently for low QA content has strategic importance to exploit the excellent agronomic value of landrace genetic resources in terms of adaptation to specific regions [20, 43], drought tolerance [44], cold tolerance [14, 45], resistance to anthracnose [37, 46], and other traits [5, 47] through crosses with sweet-seed material (i.e., cultivars or breeding lines featuring low content of QAs). Operationally, the selection of inbred lines for low total QA content can exploit rapid screening methods based on (a) Dragendorff paper test (a colorimetric method exploiting a reaction between QAs and the potassium bismuth iodide reactive) applied to adult plants [40], or (b) the fluorescence exhibited by bitter seeds when evaluated under an ultra-violet light (a quick test that lacks high discrimination power probably due to interfering substances such as flavonoids) [48]. In addition, molecular markers tagging the *pauper* locus [49–51] and other two loci [21] that are associated with high or low QA content have been developed, and further developments may derive from research on candidate genes [52]. However, the greatest challenge for breeders is selecting for very low QA content (ideally < 200 mg/kg) within broadly sweet-seed material (usually possessing the *pauper* locus), also in view of the high costs of chemical analyses. The content of QAs can be influenced by several environmental factors [26, 53] but is not affected largely by GEI based on the absence of GEI effects in a large white lupin collection evaluated across German environments [21] and the distinctly smaller size of the GEI variance component relative to the genotype variance component for narrow-leaf lupin in Australia [54] and white lupin in Italy [55].

Plant breeding for traits that are genetically complex and/or difficult to assess could become more cost-efficient by genomic selection (GS), which combines phenotyping and genotyping data of a genotype sample representing a reference population to define a statistical model for prediction of breeding values in future plant selections [56, 57]. This avenue became practically feasible after the development of next generation sequencing techniques, such as genotyping-by-sequencing (GBS) [58], that allow to genotype large germplasm sets by thousands of single nucleotide polymorphism (SNP) markers at a relatively low cost. The ability of GBS to generate thousands of polymorphic SNP markers for white lupin genetic analyses was confirmed [50]. Pioneer GS studies were encouraging for white lupin breeding. Predictive ability values (as Pearson’s correlation between predicted and observed values) were in the range of 0.40–0.60 for grain yield of landrace genotypes or breeding lines [59, 60], in the range of 0.56–0.81 for tolerance to anthracnose [21, 61], and in the range of 0.40–0.65 for various morphophysiological traits [62, 63]. They achieved 0.65 for seed protein content, and 0.75 for total QAs across material with bitter or sweet seed [21]. Genome-enabled predictions developed for landrace germplasm could also be useful to identify promising genetic resources within the large number of accessions held in germplasm collections, which exceed 6300 just considering the main white lupin collections [64] and cannot be thoroughly evaluated due to limited budgets.

This study focused on a genetically broad reference population of sweet-seed breeding lines originated from crosses of four elite landrace accessions with each of four elite sweet-seed varieties or breeding lines, and a second reference population including landrace genotypes of worldwide origin. The breeding lines were preliminarily selected for low alkaloid content provided by parents possessing the *pauper* locus. Our study aimed to support the improvement of white lupin for key seed quality traits, by assessing: (a) the extent of genetic variation in both reference populations, and that of GEI in different environments of Italy and Chile in the breeding line population, for protein content, oil content, and individual seed weight; (b) the extent of genetic variation for total QAs and 13 individual QAs in the breeding line population; (c) the degree of polygenic control and major relevant genomic areas for the target traits through a genome-wide association study (GWAS), and the consistency of GWAS results across the two reference populations and the two cropping regions; and (d) the predictive ability of genome-enabled models for the target traits, envisaging also inter-population and inter-environment prediction scenarios for the traits that were phenotyped in different reference populations and cropping regions.

## Results

### Genetic variation and genotype × environment interaction

Information on the extent of genetic variation among breeding lines or landrace genotypes (expressed as range values and genetic coefficient of variation) and broad-sense heritability in each evaluation environment for individual seed weight, protein content, oil content, and total QAs is reported in Table 1. We observed significant variation (*P* < 0.001) for every trait in any environment. Lodi (Italy) tended to display lower mean value and greater genetic variation than Temuco (Chile) for seed weight and protein content (Table 1). Landrace germplasm, compared with breeding line material, displayed much larger genetic variation for seed weight (from 0.142 to 0.845 g) and protein content (from 30.0 to 44.6%) but comparable variation for oil content (for which no landrace or breeding line genotype exceeded 11.6%). The population of breeding lines exhibited substantial variation for all traits, with some genotypes consistently exceeding 0.5 g seed weight and 39% protein content (Table 1). On the negative side, the variation of breeding lines for total QAs determined by our gas chromatography-mass spectrometry (GC/MS) method revealed a maximum value of 990 mg/kg and only about 24% of the genotypes with values below the optimal threshold of 200 mg/kg. However, we found genetic variation (*P* < 0.001) for any of the 13 QAs. On average, lupanine represented nearly 62% of the total QAs, followed by 13α-hydroxylupanine (9.6%), 13α-angeloyloxylupanine (8.4%), five QAs of which the relative average contribution ranged between 2.5% and 3.6% (angustifoline, N-methylalbine, α-isolupanine, ammodendrine, and 13α-tigloyloxylupanine), and five additional QAs of which the individual relative contribution did not exceeded 1.5% (multiflorine, tetrahydrorhombifoline, 17-oxolupanine, 13-hydroxymultiflorine, and albine) (Table 2). Broad-sense heritability values were high (> 0.7) for every trait except protein content in Temuco (Table 1) and two minor alkaloids (multiflorine and 17-oxolupanine; Table 2).

**Table 1:** Mean and range values, broad-sense heritability on a genotype mean basis (*H*^*2*^), and genetic coefficient of variation (*CV*_*g*_), for seed quality traits of sets of white lupin genotypes belonging to two reference populations evaluated in Lodi (Italy) or Temuco (Chile)

Trait	Reference population	Environment	No. of genotypes	Mean value	Range values	*H* ^2^	*CV*_*g*_ (%)^a^
Individual seed weight (g)	Breeding lines	Lodi 2019	171	0.370	0.212–0.644	0.989	19.6
Individual seed weight (g)	Breeding lines	Temuco 2020	141	0.490	0.339–0.733	0.988	17.5
Individual seed weight (g)	Breeding lines	Temuco 2021	141	0.407	0.313–0.554	0.921	12.8
Individual seed weight (g)	Landrace accessions	Lodi 2018	374	0.340	0.142–0.845	0.986	27.5
Protein content (%)	Breeding lines	Lodi 2018	174	36.0	29.0–42.1	0.719	4.8
Protein content (%)	Breeding lines	Lodi 2019	174	36.0	31.0–39.5	0.761	3.6
Protein content (%)	Breeding lines	Temuco 2020	141	37.8	34.1–41.7	0.457	2.6
Protein content (%)	Breeding lines	Temuco 2021	141	36.7	32.9–39.8	0.359	2.4
Protein content (%)	Landrace accessions	Lodi 2018	163	38.1	30.0–44.6	0.905	6.6
Oil content (%)	Breeding lines	Lodi 2018	174	9.4	7.3–11.6	0.852	8.2
Oil content (%)	Breeding lines	Lodi 2019	174	8.6	6.6–10.7	0.919	10.3
Oil content (%)	Breeding lines	Temuco 2020	141	9.1	7.5–11.4	0.923	7.9
Oil content (%)	Breeding lines	Temuco 2021	141	8.5	6.5–10.9	0.919	8.0
Oil content (%)	Landrace accessions	Lodi 2018	163	8.4	6.4–10.9	0.949	10.6
Total quinolizidine alkaloids (mg/kg)^b^	Breeding lines	Lodi 2019	142	338.3	94.9–990.4	0.964	51.0

^a^ Genetic variance different from zero at *P* < 0.001 for every trait

^b^ Relative to 13 alkaloids reported in Table 2

**Table 2:** Mean value (mg/kg) and proportion, range values, broad-sense heritability on a genotype mean basis (*H*^*2*^), and genetic coefficient of variation (*CV*_*g*_), for 13 seed quinolizidine alkaloids of 142 white lupin breeding lines evaluated in Lodi (Italy)

Alkaloid compound	Mean value	Mean proportion (%)	Range values	*H* ^2^	*CV*_g_(%)^a^
Lupanine	208.1	61.6	38.5–651.3	0.966	60.3
13α-hydroxylupanine	32.5	9.6	0.0–139.1	0.922	80.0
13α-angeloyloxylupanine	28.5	8.4	0.0–126.6	0.943	76.1
Angustifoline	12.2	3.6	1.2–45.9	0.903	66.2
N-methylalbine	12.0	3.6	1.0–62.5	0.934	86.9
α-isolupanine	11.3	3.3	2.8–29.2	0.928	49.6
Ammodendrine	11.2	3.3	3.0–27.5	0.710	42.3
13α-tigloyloxylupanine	8.7	2.6	0.0–28.6	0.876	61.5
Multiflorine	5.1	1.5	1.0–29.5	0.677	76.0
Tetrahydrorhombifoline	4.7	1.4	1.0–17.3	0.719	48.1
17-oxolupanine	2.1	0.6	0.0–13.4	0.526	54.7
13-hydroxymultiflorine	1.2	0.4	0.0–7.6	0.958	102.8
Albine	0.8	0.2	0.0–5.3	0.973	157.3

^a^ Genetic variance always different from zero at *P* < 0.001

Significant (*P* < 0.01) GEI for seed weight, protein content and oil content of breeding lines occurred across all possible pairs of evaluation environments, except for protein content across two cropping years in Temuco (Table 3). The extent of GEI according to estimates of genetic correlation for genotype response across environments was very high for protein content across Italian and Chilean environments (*r*_*g*_ < 0.25), moderately high for oil content across Italian and Chilean environments (0.47 < *r*_*g*_ < 0.66), and modest or nil (*r*_*g*_ >0.76) for protein and oil content across cropping years within the same region and for seed weight across regions or across years within each region (Table 3). In Italy, low GEI across years occurred despite the adoption of late-winter sowing in the first year and autumn sowing in the second year.

**Table 3:** Genotype × environment interaction (GEI) *P* level, and genetic correlation (*r*_*g*_), for seed quality trait response of white lupin breeding lines across pairs of evaluation environments in Lodi (Italy) or Temuco (Chile)

Trait	Environments	No. of genotypes	GEI *P*^a^	*r*_g_ ± SE^b^
Individual seed weight	Temuco: 2020 vs. 2021	141	**	0.949 ± 0.013 **
Individual seed weight	Temuco: 2020 vs. Lodi 2019	131	**	0.790 ± 0.034 **
Individual seed weight	Temuco: 2021 vs. Lodi 2019	131	**	0.805 ± 0.036 **
Protein content	Lodi: 2018 vs. 2019	174	**	0.766 ± 0.065 **
Protein content	Temuco: 2020 vs. 2021	141	NS	1.00 ± 0.198 NS
Protein content	Lodi 2018 vs. Temuco 2020	140	**	0.242 ± 0.130 **
Protein content	Lodi 2018 vs. Temuco 2021	140	**	0.232 ± 0.137 **
Protein content	Lodi 2019 vs. Temuco 2020	140	**	0.102 ± 0.129 **
Protein content	Lodi 2019 vs. Temuco 2021	140	**	0.194 ± 0.133 **
Oil content	Lodi: 2018 vs. 2019	174	**	0.955 ± 0.022 *
Oil content	Temuco: 2020 vs. 2021	141	**	0.869 ± 0.029 **
Oil content	Lodi 2018 vs. Temuco 2020	140	**	0.653 ± 0.059 **
Oil content	Lodi 2018 vs. Temuco 2021	140	**	0.486 ± 0.075 **
Oil content	Lodi 2019 vs. Temuco 2020	140	**	0.613 ± 0.060 **
Oil content	Lodi 2019 vs. Temuco 2021	140	**	0.473 ± 0.073 **

^a^ NS, *, ** = not significant, and significant at *P* < 0.05 and *P* < 0.01, respectively

^b^ SE, standard error; NS, *, ** = not different from unity, and different at *P* < 0.05 and *P* < 0.01, respectively

We compared the known characteristics of the eight parents of the breeding lines (four landrace and four sweet-seed genotypes) with that of their progeny lines obtained by computing the average value of the breeding lines belonging to each of the 16 crosses according to best-linear unbiased prediction trait values in each evaluation environment (Additional file 1: Table S1) and then averaging the trait values across the relevant evaluation environments and the parents of each cross. The ranking for mean progeny value of the parents was highly consistent with that of the parent genotypes, whenever there was available information on the parent characteristics. This was the case, in particular, for (a) the seed weight, where the progenies of the four parental landraces ranked in perfect agreement with the value of the parent genotypes, (b) the seed protein content, where the progeny lines of Arsenio displayed higher mean protein content in agreement with the higher protein content of this genotype compared with Lucky and MB-38, and (c) the total QA content of the sweet-seed parents on the one hand (with lowest QA content displayed by the progeny lines of the parent with lowest QA content) and the bitter-seed parents on the other hand (with highly consistent ranking of the progenies values with those of the four parental landraces) (Table 4). The QA amount of the parent genotypes ranged from 67 mg/kg for Lucky to 37,321 for the landrace Gr56.

**Table 4:** Average value of seed quality traits exhibited by white lupin breeding lines as a function of their parent genotype, for lines issued by crosses of each of four elite landrace accessions with each of four elite sweet-seed cultivars or breeding lines (see supplementary Table 1 for values of breeding lines in individual environments used to compute mean parent genotype values)

Parent^a^	Individual seed weight (g)^b^	Protein content (%)^c^	Oil content (%)	Total quinolizidine alkaloids (mg/kg)^d^
Gr56 (LA)	0.388	36.55	8.89	432.2
LAP123 (LA)	0.494	36.55	8.60	239.9
La246 (LA)	0.418	36.46	9.25	295.0
La646 (LA)	0.388	36.77	8.89	427.2
Arsenio (SL)	0.388	36.74	9.39	392.6
L27PS3 (SL)	0.494	36.74	8.78	337.3
Lucky (SL)	0.418	36.48	8.47	264.5
MB-38 (SL)	0.388	36.37	8.98	399.8

^a^ LA, landrace accession; SL, sweet-seed modern line or cultivar

^b^ LAP123 > La246 > Gr56 and La646 in a prior evaluation of parents [20]

^c^ Arsenio > MB-38 > Lucky in a prior evaluation of parents [87] (in which Arsenio corresponds to Line 7–50)

^d^ Lucky exhibited particularly low values in a prior study [12] and in the current study (67 mg/kg vs. about 250 mg/kg for L27PS3 and Arsenio, and 292 mg/kg for MB-38). The bitter-seed parent genotypes ranked in the order Gr56 > La646 > La246 > LAP123 in this study, with values ranging from 37,321 to 21,229 mg/kg

We investigated the phenotypic correlations between seed weight, protein content and oil content of breeding lines and landrace genotypes using best-linear unbiased prediction values, which, for breeding lines, were previously averaged across environments. The correlations were always low (*r* < 0.35) and tended to be inconsistent across the two reference populations (Table 5). The relationship of seed weight with oil content tended to be negative in breeding lines and positive in landrace material, whereas a negative correlation between protein and oil contents emerged only for landrace genotypes. Higher total content of QAs in breeding lines was associated negatively with seed weight and positively with protein and oil content (Table 5). Additional correlation results reported in Additional file 2: Table S2 aimed to verify whether a lower content of total QAs and/or its major component lupanine occurred in parallel for all the QAs. Total QA content exhibited moderate to high correlation with all QAs except multiflorine (a minor QA in our material). However, lupanine showed moderate to fairly low correlation (*r* ≤ 0.40) with 13α-hydroxylupanine, 13α-angeloyloxylupanine, angustifoline, and N-methylalbine (Additional file 2: Table S2), indicating some inconsistency in the pattern of co-variation of the most-represented QAs.

**Table 5:** Correlations between seed quality traits of white lupin genotypes belonging to breeding line (above diagonal) or landrace genotype (below diagonal) references populations, based on best-linear unbiased prediction values (which were previously averaged across evaluation environments for breeding lines). See Table 1 for description of data sets

Trait	Individual seed weight	Protein content	Oil content	Total quinolizidine alkaloids
Individual seed weight	–	0.06 NS	–0.34 **	–0.43 **
Protein content	0.30 **	–	0.03 NS	0.40 **
Oil content	0.25 **	–0.31 **	–	0.28 **
Total quinolizidine alkaloids	–	–	–	–

NS, not significant (*P* > 0.05); **, significant at *P* < 0.01

### Genome-wide association study

**Fig. 1:**
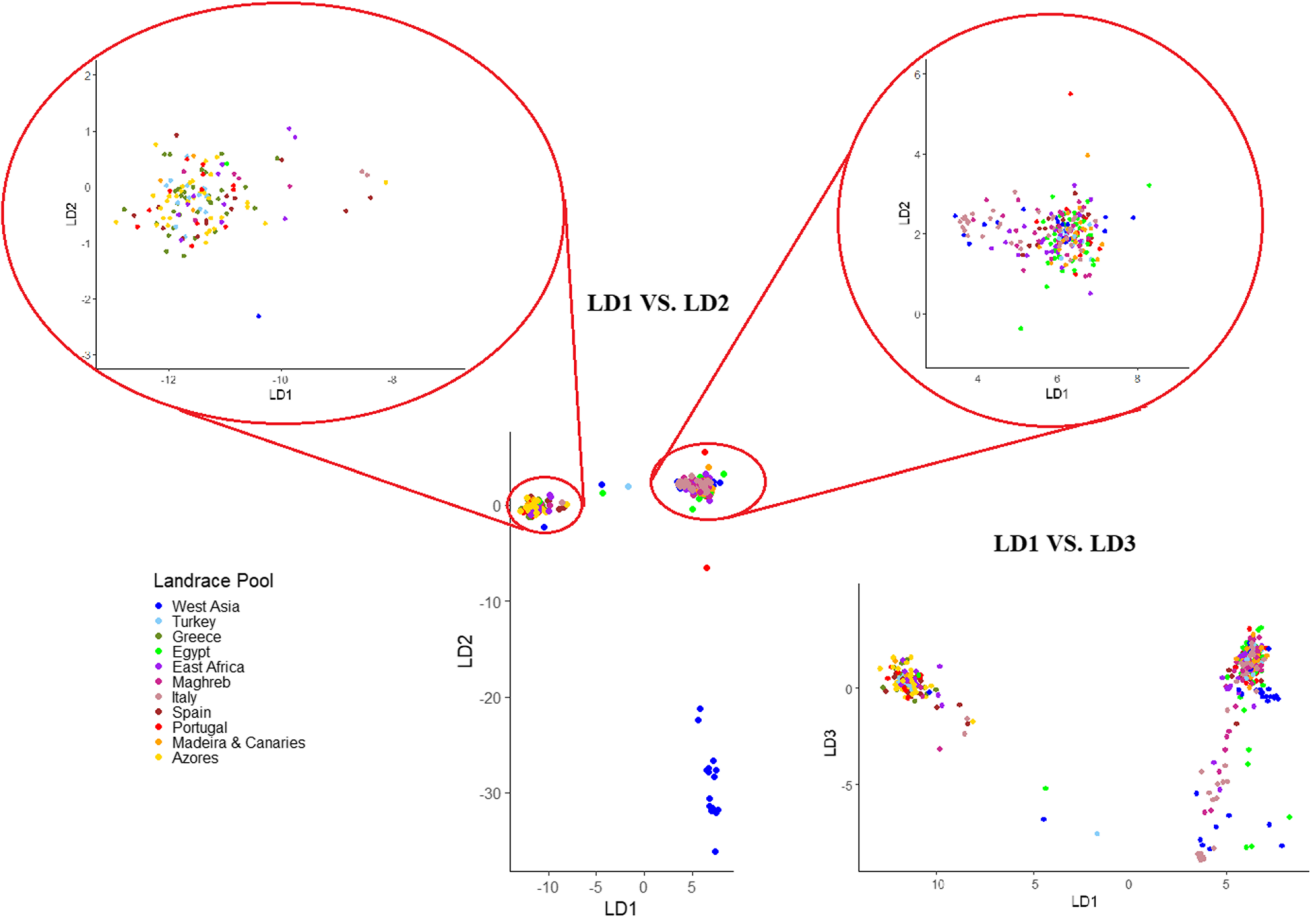
Ordination along the first three linear discriminant axes (D 1, D 2, and D 3) of a discriminant principal components analysis based on 41,116 SNP markers for 380 white lupin landrace genotypes belonging to 11 regional pools (indicated by different colors)

On average, the linkage disequilibrium (LD) reached *r*^*2*^ = 0.1 at (a) 1086 bp according to 41,116 polymorphic SNPs of the landrace reference population, and (b) 4344 bp based on 33,473 SNPs of the breeding line population. An analysis of population structure performed by discriminant principal components analysis (DAPC) preceded the GWAS. The DAPC for breeding lines detected 16 clusters corresponding to the 16 crosses that originated the lines. The DAPC for landrace material identified four clusters whose composition could be depicted according to the genotype ordination in the space of the first three linear discriminant axes (D 1, D 2, and D 3) in Fig. 1. One minor cluster featuring distinctly negative values of D 2 was entirely formed by accessions from West Asia, whereas a second minor cluster characterized by distinctly negative values of D 3 included accessions mainly from Italy, Egypt, and West Asia. The remaining landrace genotypes were grouped into two major clusters, one featuring negative D 1 values that included all the genotypes from the Azores and Greece and various accessions from the other germplasm pools (left upper corner of Fig. 1), and the other featuring positive D 1 values that comprised most of the accessions from Africa, West Asia, Italy, and Madeira and Canary islands (right upper corner of Fig. 1).

**Fig. 2:**
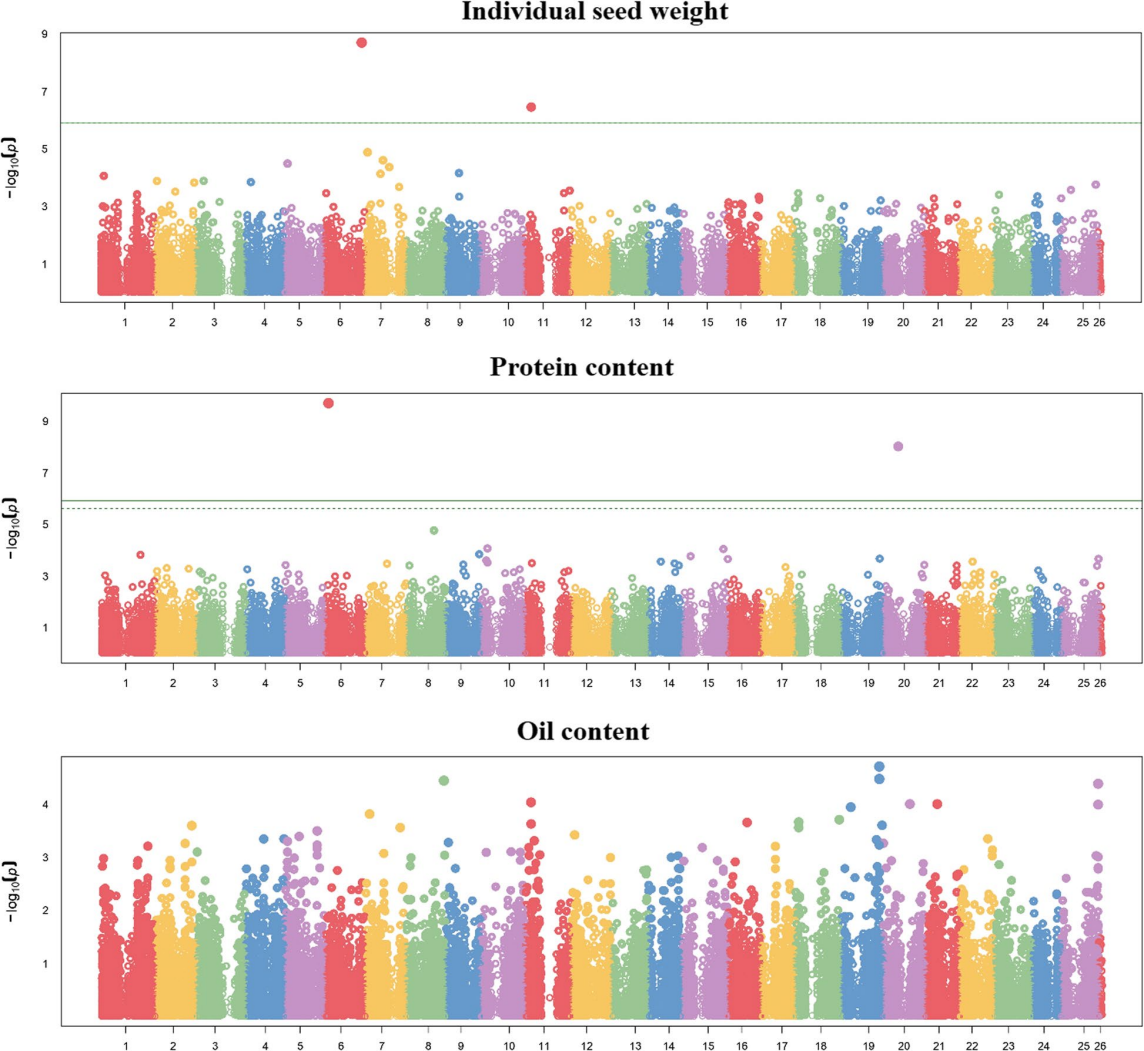
Manhattan plots showing the association scores of 41,116 SNPs (ordered along chromosomes; chromosome 26 represents scaffolds) with three white lupin seed quality traits of 374 (seed weight) or 163 (oil and protein content) landrace genotypes evaluated in Lodi (Italy). The green continuous line is Bonferroni threshold at *P* < 0.01, whereas the green dashed line (where visible) is the False Discovery Rate threshold at *P* < 0.01

Manhattan plots for the GWAS performed on landrace germplasm are reported in Fig. 2. Two significant SNPs (at *P* < 0.01 threshold) emerged for both seed weight on chromosomes 6 and 11, and protein content on chromosomes 6 and 20. No significant SNP was detected for seed oil content.

**Fig. 3:**
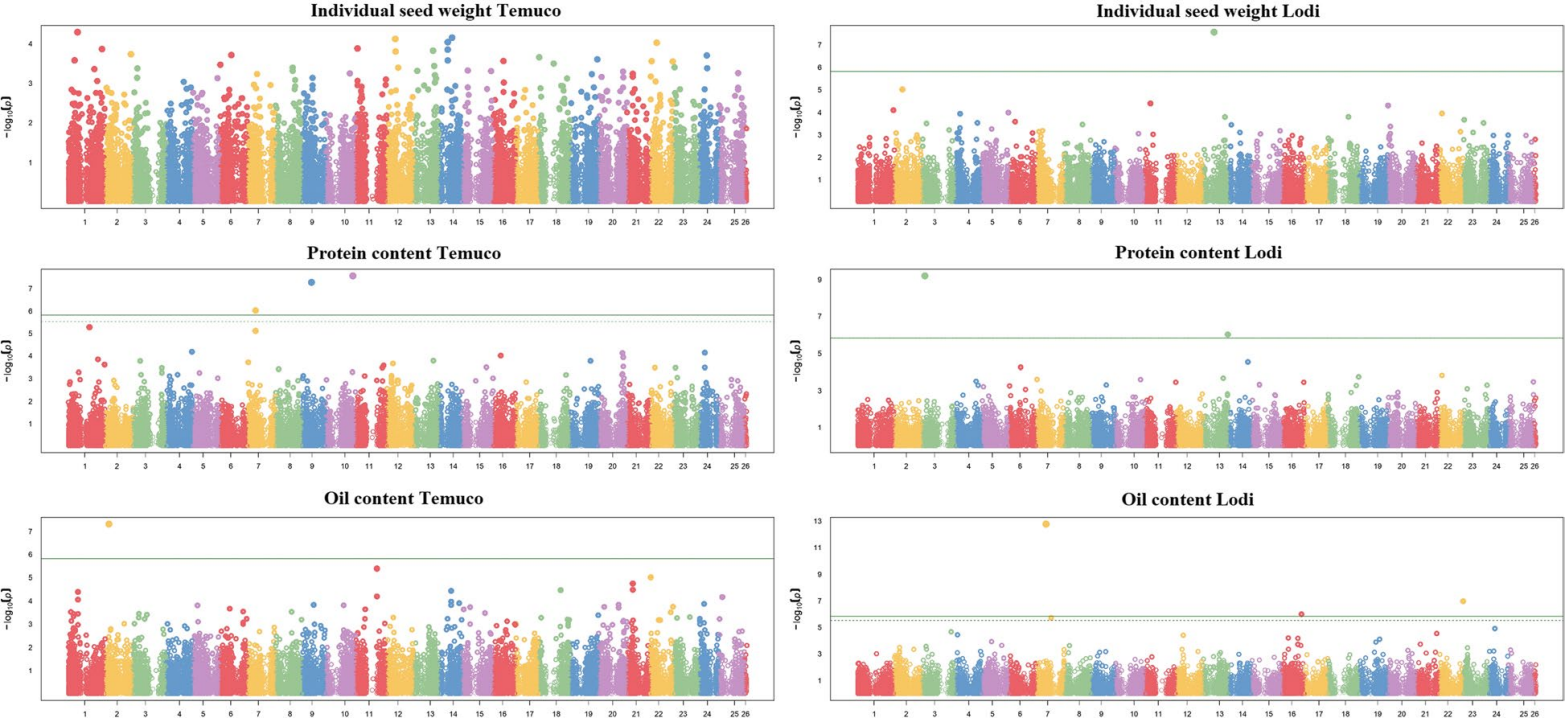
Manhattan plots showing the association scores of 33,473 SNPs (ordered along chromosomes; chromosome 26 represents scaffolds) with three seed quality traits of white lupin breeding lines evaluated in Temuco (Chile) or Lodi (Italy). The green continuous line is Bonferroni threshold at *P* < 0.01, whereas the green dashed line (where visible) is the False Discovery Rate threshold at *P* < 0.01

The GWAS for seed weight, protein content and oil content of breeding line material was performed separately for each location, owing to the relatively low *r*_*g*_ values for line response across locations (indicative of substantial genotype × location interaction) that were observed for protein and oil content. The results indicated inconsistency for significant SNPs across locations, namely: (a) three SNPs on chromosomes 7, 9 and 10 in Temuco, and two SNPs on chromosomes 3 and 13 in Lodi, for protein content; (b) one SNP on chromosome 2 in Temuco, and four SNPs of which two on different regions of chromosome 7 and the others on chromosomes 16 and 22 in Lodi, for oil content (Fig. 3). Inconsistency across locations for significant SNPs took place also for seed weight, with one SNP on chromosome 13 for Lodi and no SNP for Temuco (Fig. 3), despite the moderately high consistency of line response across locations for this trait (*r*_*g*_ ≥ 0.79; Table 3). This finding suggested that several mostly consistent QTLs with minor effects (hence, not associated to significant SNPs) were the main determinant of the genotype responses for seed weight, a hypothesis that agreed with the large number of SNPs with a modest, non-significant association score observed for this trait in the two locations (Fig. 3). Actually, a similar pattern (many SNPs with modest association score) emerged for the three traits (seed weight, protein content, and oil content) in breeding line and landrace germplasm (Figs. 2 and 3), supporting a definitely polygenic genetic architecture of these traits. Landrace and breeding line reference populations had no significant SNP in common for any of these three traits (Figs. 2 and 3).

**Fig. 4:**
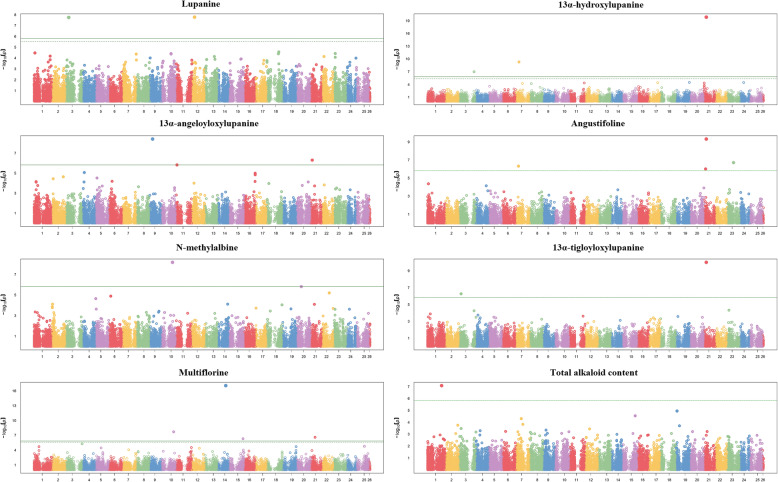
Manhattan plots showing the association scores of 33,473 SNPs (ordered along chromosomes; chromosome 26 represents scaffolds) with seed content of total quinolizidine alkaloids and seven alkaloids of 142 white lupin breeding lines. The green continuous line is Bonferroni threshold at *P* < 0.01, whereas the green dashed line (where visible) is the False Discovery Rate threshold at *P* < 0.01

The GWAS performed for the content of individual QAs in the breeding lines had higher practical interest for the most represented QAs, namely, lupanine, 13α-hydroxylupanine, and 13α-angeloyloxylupanine. We detected two significant SNPs on chromosomes 3 and 12 for lupanine, three significant SNPs on chromosomes 3, 7, and 21 (the latter with a very high association score) for 13α-hydroxylupanine, and two significant SNPs on chromosomes 9 and 21 plus one very close to significance on chromosome 11 for 13α-angeloyloxylupanine (Fig. 4). Rather unexpectedly, none of these SNPs reached significance in the GWAS for total QA content, which revealed instead a significant SNP on chromosome 1 (Fig. 4). SNPs with very high association scores were also found for multiflorine on chromosome 14 (Fig. 4) and 17-oxolupanine on chromosome 25 (Additional file 3: Fig. S1). Two SNPs on chromosome 21 resulted significant for more than one alkaloid, namely chr21_1801842 for 13α-angeloyloxylupanine and angustifoline, and chr21_2683307 for 13α-hydroxylupanine, angustifoline, and 13α-tigloyloxylupanine.

### Genome-enabled predictions

The two statistical models envisaged for GS (rrBLUP and BayesC) usually provided very similar estimates of predictive ability. Table 6 reports the predictive ability of the better-predictive of the two models for each specific trait and intra- or inter-population scenario. The intra-population, inter-environment predictive ability for breeding lines with cross validation across two cropping years in the same location, which is representative of the GS potential for region-specific breeding, was high for seed weight (0.80) and ranged from moderately high to high (from 0.41 to 0.73) for protein content and oil content (Table 6). The intra-population, inter-environment predictive ability for breeding lines with cross validation across the two regions was high for seed weight (≥ 0.64), moderate for oil content (0.36–0.40), and nil for protein content (Table 6). Predictions for the challenging inter-population, inter-environment scenario were generally better when training the GS model on landrace germplasm to predict breeding line values than the reverse (Table 6), as expected from the broader genetic variation included in the landrace reference population. It was noteworthy, in particular, the high to moderately high predictive ability (0.47–0.64) of the models constructed on landrace data to predict breeding line values of seed weight in both locations and oil content in Lodi (Table 6). Very low or nil inter-population, inter-environment predictions (≤ 0.17) emerged for protein content in any case and for oil content when training and validation data belonged to different regions (Table 6).

**Table 6:** Predictive ability according to the best-performing of two models (rrBLUP and BayesC) for four seed quality traits of two white lupin reference populations evaluated in Temuco (Chile) or Lodi (Italy) for different intra- or inter-population and intra- or inter-environment prediction scenarios. Intra-population predictions based on a 10-fold cross-validation scheme, averaging the results of 10 repetitions. See Table 1 for description of data sets

Prediction scenario	Training set	Validation set	Individual seed weight	Protein content	Oil content	Total quinolizidine alkaloids
Intra-pop., inter-env.	Lines in Temuco^a^	Lines in Temuco^a^	0.80	0.54	0.55	–
Intra-pop., inter-env.	Lines in Lodi^a^	Lines in Lodi^a^	–	0.41	0.73	–
Intra-pop., inter-env.	Lines in Temuco^b^	Lines in Lodi^b^	0.64	–0.01	0.40	–
Inter-pop., inter-env.	Lines in Lodi^b^	Lines in Temuco^b^	0.73	–0.02	0.36	–
Inter-pop., inter-env.	Landraces in Lodi	Lines in Lodi^b^	0.47	0.06	0.51	–
Inter-pop., inter-env.	Lines in Lodi^b^	Landraces in Lodi	0.22	0.10	0.21	–
Inter-pop., inter-env.	Landraces in Lodi	Lines in Temuco^b^	0.64	0.17	–0.13	–
Inter-pop., inter-env.	Lines in Temuco^b^	Landraces in Lodi	0.31	0.04	0.04	–
Intra-pop., intra-env.	Lines in Temuco^c^	Lines in Temuco	0.84	0.66	0.68	–
Intra-pop., intra-env.	Lines in Lodi^c^	Lines in Lodi	0.75	0.58	0.80	0.66
Intra-pop., intra-env.	Landraces in Lodi	Landraces in Lodi	0.59	0.49	0.49	–

^a^ Using different cropping years by turns as training or validation set, and averaging the results of the two assessments

^b^ Using data previously averaged across cropping years

^c^ Averaging results across cropping years, in case of multi-year assessment

Intra-population, intra-environment predictions (based on intra-environment cross validation) were of special interest for total QA content, which was evaluated just for one year in Lodi. We found high predictive ability in this case (0.66; Table 6). Intra-population, intra-environment prediction ability values for seed weight, protein content and oil content of landrace germplasm (also assessed in single-year experiments in Lodi) were moderately high (0.49–0.59; Table 6). These predictions, compared with those for inter-population, inter-environment scenarios based on GS model training on landrace germplasm to predict breeding line values, featured similar or just slightly higher values for seed weight of lines evaluated in Lodi or Temuco and oil content of lines evaluated in Lodi (Table 6), indicating the high reliability of the genome-enabled breeding line prediction based on the landrace training set in these cases. For breeding lines, the comparison of intra-population, intra-environment predictions (expectedly too optimistic) with intra-population, inter-environment predictions (more realistic for regional breeding) indicated a modest drop of predictive ability in the latter scenario for all traits (seed weight; protein content; oil content) within each region (Table 6), in agreement with the high genetic correlation for genotype responses across cropping years within the same region (Table 3).

## Discussion

We observed large genetic variation for seed weight, protein content and oil content, not only for the reference population of world landrace genotypes, which was expected to maximize the crop genetic variation, but also for the reference population of breeding lines. This latter population may represent well a breeder’s genetic base issued by crosses among a relatively small set of elite sweet-seed and bitter-seed parent genotypes. Using one landrace parent with large seed resulted in some breeding lines with seed weight consistently above 0.50 g. For oil content, however, achieving the value of about 17–18% that is normally adopted for industrial exploitation may be challenging for breeders on the basis of our highest observed value of 11.6%. While values of 8–11% are considered ordinary for the species [14, 65], a maximum value of 14.1% was reported in a world collection of 371 accessions [66]. Based on our results, selecting for seed weight, protein content and/or oil content would be favoured by: (a) generally high broad-sense heritability in evaluation experiments (which was relatively low only for seed protein content in Chile); (b) high consistency between parent and progeny values, as observed for seed weight and protein content; (c) low GEI across cropping years in the same location (which is relevant for region-specific breeding) and, for seed weight, low GEI even across locations of two distant regions, according to genetic correlation results; and (d) absence of high inverse phenotypic correlations between traits. On the other hand, GEI across regions was very high for protein content, and moderately high for oil content, based on genetic correlations. The very high GEI for protein content (with across-region correlations in the range of 0.10–0.24) was unexpected and suggested the presence of region-specific environmental factors contributing to the trait expression of each genotype (without ruling out some contribution to across-location GEI for protein and oil content of the different NIRS-based prediction equations used for the two regions and, for protein content, the different methods of chemical analysis upon which the equations were based). On the whole, our results confirm earlier findings for white lupin relative to: (a) high broad-sense heritability reported for seed weight, protein content and oil content in Australia [67] and protein content in Germany [21]; (b) the modest GEI across sites of the same region reported for protein and oil content in Spain [68] and seed weight and/or oil content in Italy [12, 55]; and (c) the low GEI for seed weight across locations of different European regions [20]. Relatively low GEI was also reported for seed weight, protein content and oil content of narrow-leaf lupin lines across environments of Australia [54, 69] or Germany [70]. A negative correlation of protein content with oil content, which we observed in landrace genotype material but not in breeding lines, emerged for white lupin or narrow-leaf lupin in some studies [68, 70] but not in others [67] without being very high in any case, probably because of the relatively low amount of the oil component in the seed. Indeed, protein content displayed a high negative correlation with the main seed component, namely, non-starch polysaccharides [70]. The currently inconsistent correlation of seed weight with oil content reflects inconsistent results from earlier reports [67, 70].

In white lupin germplasm collections largely including bitter-seed germplasm, lupanine was the main QA compound (70–76% averaged across entries), followed by 13α-hydroxylupanine (around 8%), albine (4–15%), and multiflorine (3–5%) [71, 72]. In our study on sweet-seed lines possessing the *pauper* locus, lupanine remained the predominant QA (nearly 62% averaged across entries) followed by 13α-hydroxylupanine (9.6%), 13α-angeloyloxylupanine (8.4%), and 10 other QAs (including albine and multiflorine) of which the average individual contribution did not exceed 3.6%. Actually, 13α-angeloyloxylupanine was not analyzed in some studies [71, 72], while being reported in other studies [36, 73]. The *pauper* locus is known to reduce drastically the content of total QAs by affecting an early pathway of their biosynthesis from the amino acid lysine [26, 39]. The effect of this locus would not be expected to alter dramatically the relative composition of the QAs [40, 42], but some changes may occur, as indicated by the very low average content of albine and multiflorine in our breeding lines. Likewise, a complete absence of albine was reported for some sweet-seed white lupin cultivars in Australia [65]. Albine and multiflorine are the product of a secondary QA biosynthetic pathway that is alternative to that for lupanine and 13α-hydroxylupanine (Additional file 4: Fig. S2), and the drastic reduction of their relative content in broadly sweet-seed germplasm compared with landrace material might be due to a particularly strong effect of the *pauper* locus on this secondary pathway. The observed fairly modest correlation of lupanine with 13α-hydroxylupanine and angustifoline could be attributed to trade-offs for two alkaloids that derive directly from lupanine (Additional file 4: Fig. S2). The relatively high average content of 13α-hydroxylupanine in our lines is positive, considering that this compound and 13α-tigloyloxylupanine (little represented in our material) are less dangerous than other QAs for human and animal feeding [53]. Anyway, QA safety thresholds refer to the total content of QAs.

Despite the preliminary selection for low total QA content performed on F_3_ and F_4_ individual seeds by two methods (fluorescence, and spectrophotometer-based determination), excessive total QA content was a widespread problem in our breeding lines, with only about 24% of the lines with a value below 200 mg/kg. Although quite high, the maximum value of 990 mg/kg observed in our lines is lower than that reported for some elite lines from another breeding program [33]. A trend of environments in northern Italy to enhance the white lupin QA content relative to Mediterranean-climate Italian environments [55], and the extensive identification of QA compounds by our GC/MS method, may have contributed to the relatively high QA content of our breeding lines. Anyway, even the line with highest total QA content showed much lower QA content than that of the least bitter landrace parent (which exceeded 21,000 mg/kg), supporting the usefulness of the combination of the two rapid screening methods adopted for our early selection of broadly sweet-seed lines. Our findings confirm the crucial importance for breeders to exploit the substantial genetic variation for total QAs occurring among breeding lines that possess the *pauper* locus. The high consistency between parent and progeny data observed for both sweet-seed and bitter-seed parents is comforting, considering the complex genetic control of this trait suggested by earlier studies [40, 41], and emphasizes the interest of identifying sweet-seed and landrace parent genotypes also on the ground of their QA content. The usefulness for GWAS and genomic selection of our phenotypic data is supported by the very high broad-sense heritability (> 0.96) and the substantial range of variation for total QA content expressed by our sweet-seed (67–292 mg/kg) and bitter seed parent genotypes (21,229 − 37,321 mg/kg), which is consistent with the overall variation reported for a large germplasm collection (about 50 − 36,000 mg/kg) [21].

The slower average LD decay in the breeding line reference population compared with the landrace genotype population is consistent with the lower number of meiotic events that affected the former population compared with the latter. The breeding line population exhibited faster LD decay than in a previous assessment [60, 63], probably because the currently greater number of lines and SNP markers allowed for greater precision in the reconstruction of the LD decay pattern. The observed LD decay for landrace germplasm was somewhat slower than in [21] and faster than in [74], these two studies including also modern germplasm besides landrace accessions. In absolute terms, however, all of these studies indicated faster rates of LD decay in white lupin relative to other grain legume species such as narrow-leafed lupin, pea, common bean, and soybean [52, 74]. Fast LD decay hindered the identification of significant SNPs and may have contributed, possibly along with rare alleles that were absent in the breeding line population, to inconsistency for significant SNPs across the two reference populations.

While the population structure of the breeding lines reflected the crosses that originated them, the DAPC results for landrace material indicated a weak population structure and a fairly loose relationship with the geographic origin of the material, in agreement with earlier results [52, 74]. The distinctness of several accessions from West Asia, and the classification of all genotypes from Greece and Azores in a common cluster despite the geographic distance of these germplasm pools, agree with earlier results based on DArT-seq markers [75]. The Greek and Azorean germplasm pools share a late phenology [76], and phenology contributed importantly to population structure in an earlier study [75].

A polygenic control of our major target traits (seed weight; protein content; oil content; total QA content) was supported by various GWAS results (i.e., several different significant SNPs for the individual QAs; several minor, non-significant peaks in Manhattan plots for the other three traits; inconsistency of significant SNPs along with high genetic correlation across locations, for seed weight). Such a genetic architecture encourages the development of GS models, to account conveniently for the effect of many minor genes in linkage with the SNPs. Seed weight resulted the most predictable trait, with high predictive ability (0.64–0.73) for intra-population prediction across contrasting locations, and high to moderately high predictive ability (0.47–0.64) even for the challenging scenario of inter-population, inter-environment predictions based on model training on the genetically broader landrace genotype population aimed to predict breeding lines. The reliability of the GS model constructed on the landrace training set for inter-population, inter-environment prediction was reinforced by its modest or nil drop of predictive ability compared with intra-population, intra-environment prediction based on the same model. This result occurred also for oil content limitedly to Lodi, which was the site that generated the GS model training data. Seed weight proved to be well-predictable by genomic selection also in other grain legumes [77–79], and a GS model for pea seed weight trained on landrace germplasm showed a predictive ability for breeding lines comparable with the current one [80]. The last study indicated a low but not negligible predictive ability for a model constructed on landrace data also for prediction of pea protein content of breeding lines, in contrast with the currently poor predictive ability of such a model for protein content of white lupin breeding lines. A major specificity of genomic areas associated with protein content in different reference populations was supported by the marked inconsistency for significant SNPs across the current landrace and breeding line populations and between each of them and the population in an earlier study [21]. Importantly, however, protein content and oil content showed moderately high inter-environment predictive ability (> 0.40) for region-specific breeding when using GS models constructed and applied on the same reference population, including Lodi, in which the two cropping years adopted a different sowing season. This result was favoured by low GEI for these traits across years within each region. The crucial impact of GEI on GS predictions was highlighted by the close relationship between intra-population, inter-environment predictive ability across the two regions (high for seed weight, moderate for oil content, and nil for protein content; Table 6) and the extent of GEI across locations (low for seed weight, moderately high for oil content, and very high for protein content; Table 3). A GS predictive ability value around 0.75 was reported for protein content of a white lupin germplasm collection evaluated in German environments that featured no significant GEI (by using cross-validations on entry values averaged across environments) [21].

Our study provides an unprecedented assessment of GS predictive ability for total QA content within broadly sweet-seed breeding lines expected to possess the *pauper* locus that were identified by preliminary phenotypic selection for low QA content through relatively quick methods. The observed predictive ability value of 0.66 is encouraging for GS of this trait, also in view of its genetic complexity and the cost of its chemical determination. A predictive ability value around 0.78 was reported for total QA content of a data set encompassing bitter-seed and sweet-seed material [21]. Because of its much wider range of total QA content (up to 36,000 mg/kg), that data set could not be compared with ours in terms of GS predictive ability, and was considered ill suited by the authors for assessing genomic effects in broadly sweet-seed material. Our GS model for total QA content would benefit from inter-environment validation (which was currently prevented by budget limitations) to obtain a more reliable assessment of its predictive ability. However, the modest GEI emerged for this trait in earlier studies [21, 54, 55] suggests a modest drop of predictive ability passing from intra-environment to inter-environment cross validation. The assessment of inter-population, inter-environment predictive ability aimed to apply this GS model to different breeding line populations is highly relevant, too.

Our SNP data set was suboptimal for a fine-mapping study aimed to strongly support the identification of candidate genes for marker-assisted selection (a selection approach of possibly limited value, anyway, for the current polygenic traits). A list of white lupin genes potentially associated with significant SNPs detected by GWAS on breeding lines or landrace genotypes is reported in Additional file 5: Table S3. We found two candidate genes of potential interest involved in gene expression, one for landrace genotype seed weight on chromosome 11 (Lalb_Chr11g0066211), and the other for oil content of breeding lines in Temuco on chromosome 2 (Lalb_Chr02g0145221). These genes encode putative transcription factors of families C2H2 and C3H, and were shown to play a role in seed germination [81] and oil production [82], respectively. Another candidate gene was detected on chromosome 22 (Lalb_Chr22g0361651) for oil content of breeding lines in Lodi. This gene encodes a cycloartenol synthase, which has a key role in the biosynthesis of sterols [83]. Many candidate genes of possible interest emerged for content of QAs, which are reported in yellow in Additional file 5: Table S3. These genes encode products belonging to one of the following categories: transcription factors or other proteins involved in gene expression control, oxidoreductases, light-responsive proteins, methyltransferases, and proteins involved in seed maturation. Table 7 reports the candidate genes that exhibited high -Log_10_ (*P* value) association score with content of QAs. The candidate gene Lalb_Chr21g0308981 encodes for the putative UV excision repair protein Rad23, which is responsible for repairing DNA damage caused by UV radiation and is involved in diverse cellular functions including stress response and development. Interestingly, this gene was associated with three different compounds within the same biosynthetic pathway of lupanine derivatives: angustifoline, 13*α*-hydroxy lupanine, and 13*α*-tigloyloxy lupanine (Additional file 4: Fig. S2). This finding makes it a promising candidate for future studies focusing on the accumulation of compounds along the lupanine branch, which includes the most abundant class of alkaloids in *L. albus*. The candidate gene Lalb_Chr21g0307601 was associated with angustifoline and 13α-angeloyloxylupanine within the same lupanine pathway. This gene encodes for a survival of motor neuron protein-interacting protein 1 (GEMIN2), a protein that plays a crucial role in the spliceosome assembly, a complex involved in RNA processing. The Lalb_Chr25g0282621 gene, associated with oxolupanine and coding for proteins involved in seed maturation, retains some interest because QAs appear in the seed and disappear from green tissues during the maturation process [73]. The gene Lalb_Chr03g0030451, associated with lupanine content, encodes for a root cap protein that plays important roles in plant root development and environmental interaction, including gravitropism, halotropism, hydrotropism [84]. The candidate gene Lalb_Chr14g0368811, associated with multiflorine content, encodes for a carboxylesterase that catalyzes the hydrolysis of ester bonds. The candidates genes Lalb_Chr05g0221621, Lalb_Chr10g0099251 and Lalb_Chr23g0265711, associated with multiflorine derivatives, encode for proteins involved in various cellular signalling pathways. Finally, the Lalb_Chr01g0018111 gene, associated with total QA content, encodes for a putative F-box protein, part of a large gene family involved in important biological processes [85]. A focus on genes encoding proteins involved in gene expression control is justified by the fact that most plant specialized metabolite pathways are regulated tightly at the transcriptional level [24]. The high number of significant SNPs and candidate genes, and the inconsistency between significant SNPs for total QA content and content of major individual QAs, reflect the complexity of the QA biosynthetic pattern, which is partly unknown [23, 24, 73]. This complexity limits considerably the possibility of selection based on candidate genes and emphasizes the practical interest of genomic selection.

**Table 7:** Candidate genes exhibiting high -Log_10_ (*P* value) association score with content of quinolizidine alkaloids

Significant SNP	Trait	-Log_10_ (P value)	Candidate gene	Putative protein
chr21_2683307	13α-hydroxy lupanine	**19.8**	Lalb_Chr21g0308981	UV excision repair protein Rad23
	13α-tigloylooxy lupanine	**10.0**		
	angustifoline	**9.3**		
chr25_9303017	oxolupanine	**17.4**	Lalb_Chr25g0282621	Seed maturation protein
chr03_3330272	lupanine	**7.8**	Lalb_Chr03g0030451	Root-cap
chr21_1801842	13α-angeloyloxy lupanine	**6.3**	Lalb_Chr21g0307601	survival of motor neuron protein-interacting protein 1 (GEMIN2)
	angustifoline	**6.0**		
chr14_10375114	multiflorine	**17.0**	Lalb_Chr14g0368811	carboxylesterase
chr05_6178718	13α-hydroxy multiflorine	**8.4**	Lalb_Chr05g0221621	SEL1 protein/HCP-like superfamily protein-related
chr10_14376008	N-methyl albine	**8.2**	Lalb_Chr10g0099251	1-phosphatidylinositol 4-kinase
chr23_480869	albine	**7.7**	Lalb_Chr23g0265711	DNA-binding bromodomain protein
chr01_18235431	total QAs	**7.1**	Lalb_Chr01g0018111	F-box domain protein

## Conclusions

This study can contribute to define cost-efficient selection strategies for key white lupin seed quality traits such as seed weight, protein content, oil content, and total QAs within broadly sweet-seed material. The GWAS highlighted a definite polygenic control of these traits, which supports the definition and exploitation of GS models. Our results relative to two geographically distant target regions (Italy and Chile) indicated that the region-specific selection for seed weight, protein content and/or oil content is favoured by high broad-sense heritability, high consistency between parent and progeny values, low GEI across cropping years in the same location, absence of high inverse phenotypic correlations between traits, and moderately high predictive ability of GS models that are built and applied on the same reference population. The application of GS models defined in one region for selection in a geographically distant region is (a) convenient for seed weight, (b) possible at the cost of a moderate decrease of predictive ability for oil content, and (c) inconvenient for protein content, owing to the different impact of GEI across regions on the predictive ability of these traits. The inter-population predictive ability, here considered in particular for the definition of GS models of large applicability that are constructed from data of the genetically broader reference population represented by the world collection of landrace genotypes, could conveniently be applied for selection in different breeding line populations or for identification of promising accessions in germplasm collections with respect to seed weight in different target regions and oil content limitedly to the region that generated the training data, whereas its application for protein content is not promising. Our study confirmed the absolute priority for breeders to select for sufficiently low alkaloid content, identified several significant SNPs and relevant candidate genes for individual QAs, and highlighted the interest of genome-enabled selection for low total QA content on the basis of intra-population predictions (with pending verification for inter-population and inter-environment predictions).

The implementation of GS in white lupin breeding programs is promising, when considering that (a) the same genotyping data could be used to select simultaneously for grain quality traits and other important traits associated with yielding ability in specific regions and/or tolerance to abiotic or biotic stresses [21, 59, 60, 63]; (b) the genotyping cost, currently around 60 €/sample for the adopted genotyping-by-sequencing method, may decrease in the future (e.g., by using a new molecular tool under development within the European project BELIS). With respect to selection for low total QA content, unpublished results by CREA Lodi indicate the ability of Near Infrared Spectroscopy (NIRS) to discriminate broadly sweet-seeds from bitter seeds in crosses between sweet-seed and landrace material by a non-destructive assessment performed on whole seeds, with a misclassification error < 1%. This finding and the current ones may allow to define an innovative pipeline to select for low total QA content that includes NIRS-based selection on whole seeds of early segregating material (e.g., the F_3_ generation) focusing on the effect of the *pauper* locus, followed by GS selection for very low QA content performed on promising inbred lines in advanced generations.

## Methods

### Plant material

Our study focused on two reference populations, one relative to sweet-seed inbred lines and the other relative to landrace genotypes. The breeding lines originated from crosses of each of four elite sweet-seed cultivars or breeding lines with each of four elite landrace accessions. Relevant information for parent choice was provided in other studies relative, in particular, to lime tolerance [86], drought tolerance [44], and genotype adaptation across Italian environments [20, 87]. The landrace La646 from the Canary islands, the newly-registered Italian variety Arsenio (referred to as Line 7–50 in earlier studies), the Italian landrace La246, and the French variety Lucky, were selected because of wide adaptation to climatically-contrasting and/or moisture-contrasting environments; the Moroccan breeding line L27PS3, because of good adaptation to drought stress environments; the Greek landrace Gr56 and the breeding line MB-38, because of high tolerance to low winter temperatures; and the Italian landrace LAP123, because of moderate lime tolerance. Seed quality characteristics contributed to parent choice, e.g., the high γ-conglutin content of Arsenio and Lucky, or the very large seed of LAP123. To further broaden the population genetic base, we used a different parent genotype within a landrace population for each cross with sweet-seed material (assuming that landraces are genetically heterogeneous, unlike modern cultivars). The crosses were performed in spring 2014. An off-season generation, which was performed in a greenhouse in 2014–2015, produced several hundred F_2_ seeds for each of the 16 crosses. Inbred lines were derived thereof via single-seed descent under insect-proof cages (to prevent cross-pollination) with the aim to generate a similar number of lines from each cross. Within-cross selection for low alkaloid content was performed (a) on F_3_ and F_4_ individual seeds by the fluorescence method [48], and (b) on F_4_ seed by a non-destructive test whose ability to predict the total QAs content of progeny plants is pending verification. This test, which adapted to single seeds a previously reported spectrophotometer-based method [22, 88], involved the immersion of one weighed seed in 3 mL of deionized water for 24 h, the spectrophotometer-based determination of the alkaloid content in the water by Reifer’s reagent (14% KI, 9% I_2_ in distilled water) at 830 nm using sparteine for calibration, and the expression of this content in ppm of seed dry weight. The test was used to discard material whose predicted QAs content belonged to the highest 25% quartile. The final population included 960 F_5_ inbred lines (60 per cross), of which 560 (35 per cross) were genotyped. A subset of these lines selected in similar proportion from each cross underwent phenotyping for this study using F_6_ seed. In particular, phenotyping in Italy was carried out on (a) 174 lines for seed protein content and oil content, (b) 171 lines for seed weight, and (c) 142 lines, the eight parent cultivars and the sweet-seed control cultivars Adam and Luxe, for total QAs. Phenotyping for protein and oil contents and seed weight in Chile was performed on 141 lines randomly selected out of the lines phenotyped in Italy.

The landrace reference population included genotypes sorted out of a world collection of 112 landrace accessions described in [20]. These landraces belonged to 11 regional germplasm pools representative of the main historical white lupin cropping regions, namely, Greece, Italy, Egypt, Spain, Portugal, Turkey, Azores, Maghreb (including Algeria and Morocco), East Africa (including Ethiopia, Kenya, and Sudan), West Asia (including Syria, Lebanon, Israel, and Jordan), and a last pool including Canary and Madeira islands. Each accession was represented by at least one individual genotype, envisaging more genotypes per accession in various cases because of the expected intra-population diversity (which was confirmed by the observed molecular marker diversity). Phenotyping was carried out on 374 genotypes for seed weight, and 163 genotypes for seed protein and oil contents.

### Phenotyping

The evaluation of breeding lines for seed protein content and oil content in Italy was based on seed samples obtained from plots of two field trials performed in Lodi (northern Italy; 45°19′ N, 9°30′ E). Each trial included 174 genotypes. The first trial (Lodi 2018) was sown in mid-February and harvested in early July 2018 according to a RCB design with three replications whose plots included 12 plants disposed in two rows spaced 30 cm with plants spaced 7 cm on the row. The second trial (Lodi 2019) was sown in mid-October 2018 and harvested in June 2019 using a RCB design with two replications whose plots comprised 30 plants disposed in three rows spaced 30 cm with plants spaced 7 cm on the row. The different sowing time of these trials reflected the possible sowing periods adopted for the crop in northern Italy. The latter trial also provided seed samples for evaluating seed weight and total QAs content. The eight parent genotypes were concurrently grown in 2019 to assess their total QAs content. The seed was inoculated with Vitalianz R Lupin inoculant (Cérience, Cissé, France) prior to sowing. Pre-emergence weed control was performed by applying 1.5 L/ha of Stomp Aqua (Basf Agro; Pendimentalin 38%). Mineral fertilization was incorporated into the seedbed at the rates of 27 kg/ha of N, 46 kg/ha of P_2_O_5_, and 50 kg/ha of K_2_O. The total rainfall over the crop cycle amounted to 433 mm for the former experiment and 525 mm for the latter. The autumn-sown trial featured 57 frost days and a lowest absolute temperature of − 12.0 °C. The evaluation of seed weight was based on 50 random seeds per plot and referred to dry weight after oven drying at 90 °C for four days. The assessment of protein and oil contents was based on two seed samples per plot obtained from each of the two field experiments, of which one sample included 10 random seeds collected from main stem pods and the other sample comprised 10 seeds collected from pods of the primary reproductive branch. These samples were analyzed independently and then averaged on a plot basis prior to statistical analysis.

The evaluation of seed weight, protein content, and oil content of landrace genotypes relied on seed samples issued by a greenhouse experiment sown in Lodi in October 2017 and harvested in June 2018. The experiment was designed as a randomized complete block (RCB) with two replications. Each replicate was represented by one pot of 25 cm diameter by 25 cm height filled with local sandy-loam soil and commercial peat in a 50:50 proportion, which hosted three plants of the relevant genotype derived from sown inoculated seed which were harvested in bulk. The plants were grown in moisture-favourable conditions by periodical irrigation. Seed weight was assessed on a random sample of 20 oven-dried seeds per replicate.

The evaluation of breeding lines for seed weight, protein content and oil content in Chile was based on seed samples obtained from two field trials (Temuco 2020 and Temuco 2021) carried out in the Carillanca Research Center (Araucania; 38°44′ S, 72°40′ W). The trials included 141 genotypes sown in autumn according to a RCB design with four replications. Each plot included 33 plants disposed in three rows spaced 35 cm with plants spaced 8 cm on the row. The seed was not inoculated, and no mineral fertilization was incorporated into the seedbed, according to the ordinary crop management in the region. The total rainfall over the crop cycle amounted to 929 mm in 2020 and 752 mm in 2021. The number of frost days and the lowest absolute temperature were 39 and − 3.7 °C, respectively, in 2020, and 52 and − 4.1 °C, respectively, in 2021. The dry individual seed weight was assessed on 200 random seeds per plot.

In Italy, protein and oil content were estimated by Near-Infrared Spectroscopy (NIRS) using ad hoc calibration equations constructed on the basis of chemical analysis performed on 148 samples (74 from breeding lines and 74 from landrace genotypes) according to Dumas method [89] for protein and AOAC [90] for oil as described in an earlier report [91]. NIRS was based on a NIRFlex 500 spectrometer (Büchi Italia, Cornaredo, Italy), analyzing flour samples milled by a MM400 Mixer Mill (Retsch Gmbh and Co., Germany) at 30 Hz for about 40 s and averaging three repetitions of the same sample. The optimal model for quantifying oil content was developed by dividing the spectra of chemically analyzed samples into two 50:50 subsets using the Kennard-Stone algorithm [92]. The first subset was used to determine the optimal number of latent variables through venetian blinds cross validation, while the second subset was used to externally validate the model. The best-performing model for protein content was derived using the entire sample set and applying venetian blinds cross validation with six splits and a blind thickness of five. The ratio of prediction to deviation (RPD) of the calibration equation was 3.3 for protein and 2.5 for oil, which are indicative of sufficient prediction accuracy for plant breeding [93]. In Chile, protein content was determined by NIRS according to the Russian State standards (FR.1.31.2009.06615) using the analyzer IntraLUM FT-10 (Russia). The calibration was constructed by the analysis performed by Kjeldahl method on a set of 169 accessions at Universidad Católica de Temuco and the samples provided by the manufacturer. The ratio of prediction to deviation (RPD) was 4.1 for protein. For oil content, the calibration was based on the samples provided by the manufacturer.

The extraction and quantification of QAs of the sweet-seed breeding lines and their parent genotypes was performed according to modified versions of the protocols described earlier [36, 71]. In brief, 100 mg of defatted flour was suspended in 1.2 mL of 0.1 N HCl, with sparteine (CAS 90-39-1; Extrasynthese, France) added as internal standard in an appropriate concentration and stirred at room temperature overnight. The mixture was centrifuged at 8000 g for 45 min at 4 °C, the supernatant was collected, and the solid was washed twice with 0.8 mL of 0.1 N HCl. The gathered extracts were alkalinized with 5% NH_4_OH to pH 10 − 11 and then applied onto an Extrelut NT 3 column (Merck, Darmstadt, Germany). The alkaloids were eluted with CH_2_Cl_2_ (4 × 3 mL), and the solvent was evaporated under vacuum. The residue was diluted in dichloromethane and analysed by GC/MS. Each sample was extracted in duplicate.

Total and individual alkaloid content was determined quantitatively using the internal standard methods by gas chromatography with Flame Ionization Detector (GC/FID; Perkin-Elmer Clarus 500), equipped with a capillary column Elite-5 MS (DB-5, 30 m × 0.32 mm × 0.25 μm; Perkin-Elmer, Milan, Italy). The oven temperature ramp was held at 90 °C for 2 min, increased to 300 °C at 7 °C/min and held at 300 °C for 10 min. Helium was used as the carrier gas, and the flow rate was set at 2 mL/min. The temperature of the injector was set at 300 °C, and the injection volume was 1 µL; the temperatures of the FID was set at 320 °C. The response factor of GC/FID was calculated using the ratio between the response of the internal standard (sparteine) and the response of the analyte standard lupanine (kindly provided by Prof. M. Wink). The regression coefficient between the analyte concentration and detector response was *R*^*2*^ = 0.99. The qualitative identification of the alkaloids was performed by GC/MS analyses that were carried out using a Perkin Elmer Clarus 500 GC equipped with a Clarus 500 mass spectrometer using the same capillary column and chromatographic conditions as for the GC/FID analyses (above mentioned). Mass spectra were acquired over a range of 40–400 atomic mass units (amu) at 1 scan/sec with ionizing electron energy of 70 eV and ion source at 230 °C. The transfer line was set at 300 °C, while the carrier gas was helium at 1.0 mL/min. The QAs were identified by determination of their elution time published mass spectra [36, 71], as well as by a peak-matching library search (Nist). We identified by this method 13 QAs listed in Table 2 along with their quantitative evaluation obtained by GC/FID analyses. The total content of QAs was obtained by summing up the quantitative data of the individual QAs.

### Statistical analysis of phenotyping data

For each trait in each environment, we carried out an analysis of variance (ANOVA) including the random factors genotype and block to assess the occurrence of genetic variation, and estimated genotype ($\:{S}_{G}^{2}$) and experimental error ($\:{S}_{e}^{2}$) components of variance according to a restricted maximum likelihood method in order to estimate genetic coefficient of variation (*CV*_*g*_) and broad-sense heritability (*H*^*2*^) values as:

$$CV_g\;=\;\left(S_G/\;m\right)\;\times\;100$$

$$H^2\;=\;S_G^2/\left(S_G^2+S_e^2/n\right)$$

where *S*_*G*_ is the square root of the genotype component of variance, *m* is the trait mean value, and *n* is the number of experiment replications. Broad-sense heritability values were used to calculate best linear unbiased predictions (BLUP) values of the genotypes according to [94]. BLUP values were used (a) to estimate Pearson’s phenotypic correlations between traits and to compute mean trait values of breeding lines issued by each parent genotype, after averaging genotype trait values across evaluation environments, and (b) as phenotypic data for GWAS and GS analyses. The occurrence of GEI for seed weight, protein content or oil content of breeding lines across pairs of cropping years in the same site (Lodi or Temuco) and across the two sites in different possible years was assessed by an ANOVA including the fixed factor environment and the random factors genotype and block within environment. The size of the GEI was estimated by computing the genetic correlation (*r*_*g*_) for genotype values across pairs of environments according to [95], testing each correlation for statistical difference to unity (which was indicative of inconsistent response across environments) on the ground of confidence intervals computed by multiplying standard errors by relevant *t* values [96]. All analyses of phenotyping data were carried out using SAS/STAT^®^ software [97].

### Genotyping

Genomic DNA was extracted from young leaves of F_5_ plants of each inbred line and landrace genotype using the DNeasy Plant Mini Kit (Qiagen, Milan, Italy). Nucleic acid was quantified by a Quant-iT™ PicoGreen™ dsDNA Assay Kit (P7589, Life Technologies Italia), checking its quality by 1% agarose gel electrophoresis. A trial digestion was carried out on 10% of the DNA samples using the Optizyme EcoRI restriction enzyme (25000 U, Fisher BioReagents), to compare bands of cut and uncut DNA. The reaction was performed at 37 °C for one hour and the enzyme was deactivated at 65 °C for 20 min. DNA samples were sent to The Elshire Group Ltd. laboratory (Palmerston North, New Zealand) for outsourced library preparation and sequencing. GBS data were generated according to [58] with the following changes: we used 100 ng of genomic DNA and 3.6 ng of total adapters and restricted the genomic DNA with *Ape*KI enzyme (NEB New England Biolabs, R0643L); then, the library was amplified with Kapa Taq polymerase Alpha (KAPA Library Amplification Readymix, Kapa Biosystems KK2611) by 14 PCR cycles. Sequencing was performed on a single Illumina HiSeq X lane, at 2 × 150 bp paired end. Adopting *Ape*KI as the restriction enzyme according to [58] was supported by the fact that about 60% of the white lupin genome includes repetitive DNA sequences [98], which this enzyme tends to skip.

### SNP calling procedures, data filtering, and imputation

SNP calling was performed using *Legpipe2* pipeline [99] with default settings for diploid species, with a preliminary filtering for mapping quality (MQ < 40) but not for coverage. For alignment, we used the *Lupinus albus* genome version 1.0 [98] downloaded from https://www.whitelupin.fr/. The whole set formed by landraces and lines was filtered for monomorphic markers, minor allele frequency (MAF) > 5%, missing rate per marker < 1%, missing rate per individual < 10%, and SNP heterozygosity < 30%. The MAF was computed separately for the landrace and line set due to relevant differences in allele frequency between the two datasets, while the other filtering parameters were estimated on the whole material set. This process retained 41,116 SNPs for the landraces and 33,473 SNPs for the lines. According to [100], we estimated missing data by random forest imputation [101] using the R package MissForest [102] with the configuration ntree = 100, maxiter = 10 and encoding genotypes as categorical data (factors).

### Analysis of population structure and genome-wide association study

Population structure was investigated by a discriminant analysis of principal components (DAPC) [103] performed separately for the two reference populations. The k-means clustering algorithm was used iteratively by testing cluster numbers (K) from 1 to 30 to identify the optimal value according to the local minimum of the Bayesian information criterion. DAPC was performed on the output of a principal component analysis conducted on SNP data to benefit from dimensionality reduction but keeping all the components to avoid information loss. The final DAPC was performed according to the optimal K value, which resulted equal to 4 for landrace material and 16 for breeding lines. The number of principal component (PC) axes retained for DAPC was determined by visual inspection of the plots of PC cumulative variance, whereas the number of discriminant functions used as covariates in the GWAS was defined on the basis of discriminant function eigenvalues. Accordingly, 200 and 125 PCs were considered for DAPC, and 3 and 8 discriminant functions were employed as GWAS covariates, respectively for the landrace and the breeding line set. The whole procedure was implemented by using the functions find.clusters() and dapc() from R package adegenet [104].

We estimated the LD as *r*^*2*^ value for pairwise combinations of SNPs within a 100 kb window through LD.decay() function of the R package sommer [105]. The *r*^*2*^ values were plotted against physical distance and fitted by a polynomial curve. A GWAS based on 41,116 SNPs was performed for grain protein content, oil content, and seed weight of landrace genotypes according to the Blink model [106] of the R package GAPIT [107]. Other GWAS based on 33,473 SNPs were conducted by the same method for seed weight, protein content, and oil content of the breeding lines in each location, using phenotypic data averaged across cropping years for traits subjected to multi-year evaluation. Additional GWAS for breeding lines focused on total QA content and content of 13 individual QAs recorded in Lodi. A visual examination of the quantile-quantile plots (Additional file 6: Fig. S3; Additional file 7: Fig. S4; Additional file 8: Fig. S5) indicated a proper compensation of population structure by GWAS model covariates in all cases except for the alkaloid lupanine in the breeding line set, which showed a slightly suboptimal compensation. The False Discovery Rate threshold at 1% was employed to select significant SNPs.

### Genomic selection

Genomic predictions were assessed according to two statistical models, ridge regression BLUP (rrBLUP) [56] and BayesC [108], using the R package GROAN [109]. The rrBLUP model assumes that marker effects have a common variance (which makes it particularly suitable for traits controlled by many quantitative trait loci with a small effect), whereas Bayesian models allow marker effects to have different variances [110]. Predictive ability values were computed as Pearson’s correlation between observed phenotypic values and values predicted by the model. We envisaged the following prediction scenarios: (a) intra-population, inter-environment prediction with cross validations across different cropping years of the same location (Lodi or Temuco), for traits of breeding lines subjected to within-site multi-year evaluation; (b) intra-population, inter-environment prediction with cross validations across locations for different combination of individual cropping years, for seed weight, protein content, and oil content of breeding lines; (c) inter-population, inter-environment predictions for seed weight and protein or oil content, envisaging GS model training on data of landrace germplasm in Lodi to predict breeding line values in each location or the reverse, namely, GS model training on breeding line data in either location to predict landrace genotype data (using breeding line data averaged across cropping years); (d) intra-population, intra-environment prediction, performed for each trait but considered of particular importance for traits evaluated in just one environment, such as total QA content of breeding lines and landrace germplasm traits. Ten-fold cross validations were used for each intra-population scenario.

## Supplementary Information

Supplementary Material 1.

Supplementary Material 2.

Supplementary Material 3.

Supplementary Material 4.

Supplementary Material 5.

Supplementary Material 6.

Supplementary Material 7.

Supplementary Material 8.

## Data Availability

Genotyping and phenotyping data are publicly available in the following repository Figshare: doi.org/10.6084/m9.figshare.28768163.

## Abbreviations

ANOVA: Analysis of variance
BLUP: Best linear unbiased prediction
DPCA: Discriminant principal components analysis
D: Discriminant axis
GBS: Genotyping-by-sequencing
GC/FID: Gas chromatography with Flame Ionization Detector
GC/MS: Gas chromatography-mass spectrometry
GEI: Genotype × environment interaction
GS: Genomic selection
GWAS: Genome-wide association study
LD: Linkage disequilibrium
PC: Principal component
QA: Quinolizidine alkaloid
RCB: Randomized complete block
rrBLUP: Ridge regression best linear unbiased prediction
SNP: Single nucleotide polymorphism

## References

[1] Foyer CH, Lam H-M, Nguyen HT, Siddique KHM, Varshney RK, Colmer TD, et al. Neglecting legumes has compromised human health and sustainable food production. Nat Plants. 2016;2:16112. 28221372 10.1038/nplants.2016.112

[2] Watson CA, Reckling M, Preissel S, Bachinger J, Bergkvist G, Kuhlman T, et al. Grain legume production and use in European agricultural systems. Adv Agron. 2017;144:235–303.

[3] Pilorgé E, Muel F. What vegetable oils and proteins for 2030? Would the protein fraction be the future of oil and protein crops? OCL. 2016;23:D402.

[4] Fehér A, Gazdecki M, Véha M, Szakály M, Szakály Z. A comprehensive review of the benefits of and the barriers to the switch to a plant-based diet. Sustainability. 2020. 10.3390/su12104136.

[5] Kurlovich BS. The history of lupin domestication. In: Kurlovich BS, editor. Lupins: geography, classification, genetic resources and breeding. St. Petersburg, Russia: Intan; 2002. pp. 147–64.

[6] Lucas MM, Stoddard FL, Annicchiarico P, Frías J, Martínez-Villaluenga C, Sussmann D, et al. The future of lupin as a protein crop in Europe. Front Plant Sci. 2015;6:705. 26442020 10.3389/fpls.2015.00705 PMC4561814

[7] Abraham EM, Ganopoulos I, Madesis P, Mavromatis A, Mylona P, Nianiou-Obeidat I, et al. The use of lupin as a source of protein in animal feeding: genomic tools and breeding approaches. Int J Mol Sci. 2019;20:851. 30781397 10.3390/ijms20040851 PMC6413129

[8] Boukid F, Pasqualone A. Lupine (*Lupinus* spp.) proteins: characteristics, safety and food applications. Eur Food Res Technol. 2022;248:345–56.

[9] Prusinski J. White lupin (*Lupinus albus* L.) – nutritional and health values in human nutrition – a review. Czech J Food Sci. 2017;35:95–105.

[10] Pereira A, Ramos F, Sanches Silva A. Lupin (*Lupinus albus* L.) seeds: balancing the good and the bad and addressing future challenges. Molecules. 2022;27:8557. 36500649 10.3390/molecules27238557 PMC9737668

[11] Boschin G, D’Agostina A, Annicchiarico P, Arnoldi A. The fatty acid composition of the oil from *Lupinus albus* cv. luxe as affected by environmental and agricultural factors. Eur Food Res Technol. 2007;225:769–76.

[12] Boschin G, D’Agostina A, Annicchiarico P, Arnoldi A. Effect of genotype and environment on fatty acid composition of *Lupinus albus* L. seed. Food Chem. 2008;108:600–6. 26059138 10.1016/j.foodchem.2007.11.016

[13] Edwards AC, van Barneveld RJ. Lupins for livestock and fish. In: Gladstones JS, Atkins CA, Hamblin J, editors. Lupins as crop plants. Biology, production and utilization. New York: CAB International; 1998. pp. 385–411.

[14] Papineau J, Huyghe C. Le lupin Doux Protéagineux. Paris: Editions France Agricole; 2004.

[15] Szczepański A, Adamek-Urbańska D, Kasprzak R, Szudrowicz H, Śliwiński J, Kamaszewski M. Lupin: a promising alternative protein source for aquaculture feeds? Aquac Rep. 2022;26:101281.

[16] Annicchiarico P. Adaptation of cool-season grain legume species across climatically-contrasting environments of Southern Europe. Agron J. 2008;100:1647–54.

[17] Cernay C, Pelzer E, Makowski D. A global experimental dataset for assessing grain legume production. Sci Data. 2016;3:160084. 27676125 10.1038/sdata.2016.84 PMC5037976

[18] Green AG, Oram RN. Variability for protein and oil quality in *Lupinus albus*. Anim Feed Sci Technol. 1983;9:271–82.

[19] Terigar BG, Balasubramanian S, Sabliov CM, Lima M, Boldor D. Soybean and rice bran oil extraction in a continuous microwave system: from laboratory- to pilot-scale. J Food Eng. 2011;104:208–17.

[20] Annicchiarico P, Harzic N, Carroni AM. Adaptation, diversity, and exploitation of global white lupin (*Lupinus albus* L.) landrace genetic resources. Field Crops Res. 2010;119:114–24.

[21] Schwertfirm G, Schneider M, Haase F, Riedel C, Lazzaro M, Rege-Wehling, et al. Genome-wide association study revealed significant SNPs for anthracnose resistance, seed alkaloids and protein content in white lupin. Theor Appl Genet. 2024;137:155. 38858311 10.1007/s00122-024-04665-2 PMC11164739

[22] Wink M. Quinolizidine alkaloids. In: Waterman P, editor. Methods in plant biochemistry. London: Academic; 1993. pp. 197–239.

[23] Aniszewski T. Alkaloids – Secrets of life. Amsterdam: Elsevier; 2007.

[24] Mancinotti D, Frick KM, Geu-Flores F. Biosynthesis of quinolizidine alkaloids in lupins: mechanistic considerations and prospects for pathway elucidation. Nat Prod Rep. 2022;39:1423. 35302146 10.1039/d1np00069a

[25] Wink M. Quinolizidine and pyrrolizidine alkaloid chemical ecology – A mini-review on their similarities and differences. J Chem Ecol. 2019;45:109–15. 30079442 10.1007/s10886-018-1005-6

[26] Frick KM, Kamphuis LG, Siddique KH, Singh KB, Foley RC. Quinolizidine alkaloid biosynthesis in lupins and prospects for grain quality improvement. Front Plant Sci. 2017;8:87. 28197163 10.3389/fpls.2017.00087 PMC5281559

[27] Schrenk D, Bodin L, Chipman JK, del Mazo J, Grasl-Kraupp B, Hogstrand C, et al. Scientific opinion on the risks for animal and human health related to the presence of Quinolizidine alkaloids in feed and food, in particular in lupins and lupin-derived products. EFSA J. 2019;17:5860. 10.2903/j.efsa.2019.5860 PMC7008800 32626161

[28] ACNFP. Report on seeds from narrow leafed lupin, appendix IX. London: MAFF; 1996.

[29] ANZFA. Lupin alkaloids in food. A toxicological review and risk assessment. Aust N Z Food Auth Tech Rep Ser. 2001;3:1–21.

[30] FIRAG. Risk assessment of alkaloid occurrence in lupin seed. Federal Institute for Risk Assessment Germany; 2017.

[31] Estivi L, Buratti S, Fusi D, Benedetti S, Rodríguez G, Brandolini A, et al. Alkaloid content and taste profile assessed by electronic tongue of *Lupinus albus* seeds debittered by different methods. J Food Compos Anal. 2022;114:104810.

[32] Keuth O, Humpf HU, Fürst P. Quinolizidine alkaloids in lupine flour and lupine products from the German retail market and risk assessment of the results regarding human health. Food Addit Contam Part Chem Anal Control Expo Risk Assess 2023;Apr 3:1–8. 10.1080/19440049.2023.2195954 37011027

[33] Jacob I, Feuerstein U, Heinz M, Schott M, Urbatzka P. Evaluation of new breeding lines of white lupin with improved resistance to anthracnose. Euphytica. 2017;213:236.

[34] Muzquiz M, Cuadrado C, Ayet G, de la Cuadra C, Burbano C, Osagie A. Variation of alkaloid components of lupin seeds in 49 genotypes of *Lupinus albus* from different countries and locations. J Agric Food Chem. 1994;42:1447–50.

[35] Brand TS, Brandt DA. Alkaloid content of South African lupins (*L. luteus*, *L. albus* and *L. angustifolius* species) and determination thereof by near infra-red reflectance spectroscopy. S Afr J Anim Sci. 2000;30(Suppl 1):11–2.

[36] Boschin G, Annicchiarico P, Resta D, D’Agostina A, Arnoldi A. Quinolizidine alkaloids in seeds of lupin genotypes from different origins. J Agric Food Chem. 2008;56:3657–63. 18433102 10.1021/jf7037218

[37] Zafeiriou I, Polidoros AN, Baira E, Kasiotis KM, Machera K, Mylona PV. Mediterranean white lupin landraces as a valuable genetic reserve for breeding. Plants. 2021;10:2403. 34834766 10.3390/plants10112403 PMC8619254

[38] Madelou NA, Melliou E, Magiatis P. Quantitation of *Lupinus* spp. quinolizidine alkaloids by qNMR and accelerated debittering with a resin-based protocol. Molecules. 2024;29:582. 38338327 10.3390/molecules29030582 PMC10856427

[39] Mancinotti D, Czepiel K, Taylor JL, Golshadi Galehshahi H, Møller LA, Jensen MK, et al. The causal mutation leading to sweetness in modern white lupin cultivars. Sci Adv. 2023;9:eadg8866. 37540741 10.1126/sciadv.adg8866 PMC10403207

[40] Harrison JEM, Williams W. Genetical control of alkaloids in *Lupinus albus*. Euphytica. 1982;31:357–64.

[41] Święcicki W, Górny A, Barzyk P, Gawłowska M, Kaczmarek Z. Genetic analysis of alkaloid accumulation in seeds of white lupin (*Lupinus albus* L). Genetika. 2019;51:961–73.

[42] Osorio CE, Till BJ. A bitter-sweet story: unraveling the genes involved in quinolizidine alkaloid synthesis in *Lupinus albus*. Front Plant Sci. 2022;12:795091. 35154186 10.3389/fpls.2021.795091 PMC8826574

[43] Noffsinger SL, van Santen E. Evaluation of *Lupinus albus* L. germplasm for the southeastern USA. Crop Sci. 2005;45:1941–50.

[44] Annicchiarico P, Romani M, Pecetti L. White lupin variation for adaptation to severe drought stress. Plant Breed. 2018;137:782–9.

[45] Franguelli N, Cavalli D, Notario T, Pecetti L, Annicchiarico P. Frost tolerance improvement in pea and white lupin by a high-throughput phenotyping platform. Front Plant Sci. 2024;15:1490577. 39759246 10.3389/fpls.2024.1490577 PMC11695127

[46] Adhikari KN, Buirchell BJ, Thomas GJ, Sweetingham MW, Yang H. Identification of anthracnose resistance in *Lupinus albus* L. and its transfer from landraces to modern cultivars. Crop Pasture Sci. 2012;60:472–9.

[47] Tosoroni A, Di Vittori V, Nanni L, Musari E, Papalini S, Bitocchi E, et al. Recent advances in molecular tools and pre-breeding activities in white lupin (*Lupinus albus*). Plants. 2025;14:914. 40265878 10.3390/plants14060914 PMC11945954

[48] Von Baer E, Perez I. Quality standard propositions for commercial grain of white lupin (*Lupinus albus*). In: Proceedings of the 6th International Lupin Conference. Temuco, Chile: International Lupin Association; 1991. p 158–167.

[49] Lin R, Renshaw D, Luckett D, Clements J, Yan G, Adhikari K, et al. Development of a sequence-specific PCR marker linked to the gene *pauper* conferring low-alkaloids in white lupin (*Lupinus albus* L.) for marker assisted selection. Mol Breed. 2009;23:153–61.

[50] Książkiewicz M, Nazzicari N, Yang H, Nelson MN, Renshaw D, Rychel S, et al. A high-density consensus linkage map of white lupin highlights synteny with narrow-leafed lupin and provides markers tagging key agronomic traits. Sci Rep. 2017;7:15335. 29127429 10.1038/s41598-017-15625-w PMC5681670

[51] Rychel S, Książkiewicz M. Development of gene-based molecular markers tagging low alkaloid pauper locus in white lupin (*Lupinus albus* L). J Appl Genet. 2019;60:269–81. 31410824 10.1007/s13353-019-00508-9 PMC6803572

[52] Hufnagel B, Soriano A, Taylor J, Divol F, Kroc M, Sanders H, et al. Pangenome of white lupin provides insights into the diversity of the species. Plant Biotechnol J. 2021;19:2532–43. 34346542 10.1111/pbi.13678 PMC8633493

[53] Rodés-Bachs C, Van der Fels-Klerx HJ. Impact of environmental factors on the presence of Quinolizidine alkaloids in lupins: a review. Food Addit Contam Part A Chem Anal Control Expo Risk Assess. 2023;40:757–69. 37235811 10.1080/19440049.2023.2217273

[54] Cowling WA, Huyghe C, Swiecicki W. Lupin breeding. In: Gladstones JS, Atkins CA, Hamblin J, editors. Lupins as crop plants. Biology, production and utilization. New York: CAB International; 1998. pp. 93–120.

[55] Annicchiarico P, Manunza P, Arnoldi A, Boschin G. Quality of *Lupinus albus* L. (white lupin) seed: extent of genotypic and environmental effects. J Agric Food Chem. 2014;62:6539–45. 24934884 10.1021/jf405615k

[56] Meuwissen THE, Hayes BJ, Goddard ME. Prediction of total genetic value using genome-wide dense marker maps. Genetics. 2001;157:1819–29. 11290733 10.1093/genetics/157.4.1819 PMC1461589

[57] Heffner EL, Lorenz AJ, Jannink JL, Sorrells ME. Plant breeding with genomic selection: gain per unit time and cost. Crop Sci. 2010;50:1681–90.

[58] Elshire RJ, Glaubitz JC, Sun Q, Poland JA, Kawamoto K, Buckler ES, et al. A robust, simple genotyping-by-sequencing (GBS) approach for high diversity species. PLoS One. 2011;6:e19379. 21573248 10.1371/journal.pone.0019379 PMC3087801

[59] Annicchiarico P, Nazzicari N, Ferrari B, Harzic N, Carroni AM, Romani M, et al. Genomic prediction of grain yield in contrasting environments for white lupin genetic resources. Mol Breed. 2019;39:142.

[60] Pecetti L, Annicchiarico P, Crosta M, Notario T, Ferrari B, Nazzicari N. White lupin drought tolerance: genetic variation, trait genetic architecture, and genome-enabled prediction. Int J Mol Sci. 2023;24:2351. 36768674 10.3390/ijms24032351 PMC9916572

[61] Rychel-Bielska S, Nazzicari N, Plewiński P, Bielski W, Annicchiarico P, Książkiewicz M. Development of PCR-based markers and whole-genome selection model for anthracnose resistance in white lupin (*Lupinus albus* L). J Appl Genet. 2020;61:531–45. 32968972 10.1007/s13353-020-00585-1 PMC7652745

[62] Annicchiarico P, Nazzicari N, Ferrari B. Genetic and genomic resources in white lupin and the application of genomic selection. In: Singh KB, Kamphuis LG, Nelson MN, editors. The lupin genome. Cham, Switzerland: Springer Nature Switzerland AG; 2020. pp. 139–49.

[63] Annicchiarico P, de Buck A, Vlachostergios DN, Heupink D, Koskosidis A, Nazzicari N, et al. White lupin adaptation to environments with calcareous soils: phenotypic variation and genome-enabled prediction. Plants. 2023;12:1139. 36903997 10.3390/plants12051139 PMC10005150

[64] Buirchell BJ. Cowling WA. Genetic resources in lupins. In: Gladstones JS, Atkins CA, Hamblin J, editors. Lupins as crop plants. Biology, production and utilization. New York: CAB International; 1998. p. 41–66.

[65] Petterson DS. Composition and food uses. In: Gladstones JS, Atkins CA, Hamblin J, editors. Lupins as crop plants. Biology, production and utilization. New York: CAB International; 1998. pp. 353–84.

[66] Rybiński W, Święcicki W, Bocianowski J, Börner A, Starzycka-Korbas E, Starzycki M. Variability of fat content and fatty acids profiles in seeds of a Polish white lupin (*Lupinus albus* L.) collection. Genet Resour Crop Evol. 2018;65:417–31.

[67] Green AG, Oram RN, Read BJ. Genetic variation for seed yield, protein content, oil content, and seed weight in *Lupinus albus*. Aust J Agric Res. 1977;28:785–93.

[68] Jimenez MD, Cubero JI, de Haro A. Genetic and environmental variability in protein, oil and fatty acid composition in high-alkaloid Hite lupin (*Lupinus albus*). J Sci Food Agric. 1991;55:27–35.

[69] Cowling WA, Tarr A. (2004) Effect of genotype and environment on seed quality in sweet narrow-leafed lupin (*Lupinus angustifolius* L.). Aust J Agric Res. 2004;55:745–751.

[70] Beyer H, Schmalenberg AK, Jansen G, Jürgens HU, Uptmoor R, Broer I, et al. Evaluation of variability, heritability and environmental stability of seed quality and yield parameters of *L. angustifolius*. Field Crops Res. 2015;174:40–7.

[71] Wink M, Carsten Meissner C, Witte L. Patterns of Quinolizidine alkaloids in 56 species of the genus *Lupinus*. Phytochem. 1995;38:139–53.

[72] Kroc M, Rybiński W, Wilczura P, Kamel K, Kaczmarek Z, Barzyk P, et al. Quantitative and qualitative analysis of alkaloids composition in the seeds of a white lupin (*Lupinus albus* L.) collection. Genet Resour Crop Evol. 2017;64:1853–60.

[73] Namdar D, Mulder PPJ, Ben-Simchon E, Hacham Y, Basheer L, Cohen O, et al. New analytical approach to Quinolizidine alkaloids and their assumed biosynthesis pathways in lupin seeds. Toxins. 2024;16:163. 38535829 10.3390/toxins16030163 PMC10974633

[74] Alkemade JA, Nazzicari N, Messmer MM, Annicchiarico P, Ferrari B, Voegele RT, et al. Genome-wide association study reveals white lupin candidate gene involved in anthracnose resistance. Theor Appl Genet. 2022;135:1011–24. 34988630 10.1007/s00122-021-04014-7 PMC8942938

[75] Rychel-Bielska S, Bielski W, Surma A, Annicchiarico P, Belter J, Kozak B, et al. A GWAS study highlights significant associations between a series of indels in a FLOWERING LOCUS T gene promoter and flowering time in white lupin (*Lupinus albus* L). BMC Plant Biol. 2024;24:722. 39075363 10.1186/s12870-024-05438-1 PMC11285409

[76] Annicchiarico P, Harzic N, Huyghe C, Carroni AM. Ecological classification of white lupin landrace genetic resources. Euphytica. 2011;180:17–25.

[77] Zhang J, Song Q, Cregan PB, Jiang GL. Genome-wide association study, genomic prediction and marker-assisted selection for seed weight in soybean (*Glycine max*). Theor Appl Genet. 2016;129:117–30. 26518570 10.1007/s00122-015-2614-x PMC4703630

[78] Roorkiwal M, Rathore A, Das RR, Singh MK, Jain A, Srinivasan S, et al. Genome-enabled prediction models for yield related traits in chickpea. Front Plant Sci. 2016;7:1666. 27920780 10.3389/fpls.2016.01666 PMC5118446

[79] Burstin J, Salloignon P, Chabert-Martinello M, Magnin-Robert J-B, Siol M, Jacquin F, et al. Genetic diversity and trait genomic prediction in a pea diversity panel. BMC Genomics. 2015;16:105. 25765216 10.1186/s12864-015-1266-1 PMC4355348

[80] Crosta M, Romani M, Nazzicari N, Ferrari B, Annicchiarico P. Genomic prediction and allele mining of agronomic and morphophysiological traits in pea germplasm collections. Front Plant Sci. 2023;14:1320506. 38186592 10.3389/fpls.2023.1320506 PMC10766761

[81] Cheng X, Cao J, Gao C, Gao W, Yan S, Yao H, et al. Identification of the wheat C3H gene family and expression analysis of candidates associated with seed dormancy and germination. Plant Physiol Biochem. 2020;156:524–53. 33053501 10.1016/j.plaphy.2020.09.032

[82] Taheri H. Transcriptional modulation of structural and regulatory genes involved in isoprene biosynthesis and their relevance to oil yield and menthol content in peppermint (*Mentha piperita* L.) upon MeJA and GA 3 treatments. Russ J Plant Physiol. 2019;66:503–8.

[83] Du Y, Fu X, Chu Y, Wu P, Liu Y, Ma L, et al. Biosynthesis and the roles of plant sterols in development and stress responses. Int J Mol Sci. 2022;23:2332. 35216448 10.3390/ijms23042332 PMC8875669

[84] Das KK, Mohapatra A, George AP, Chavali S, Witzel K, Ramireddy E. The proteome landscape of the root cap reveals a role for the jacalin-associated lectin JAL10 in the salt-induced endoplasmic reticulum stress pathway. Plant Commun. 2023;4:100726. 37789617 10.1016/j.xplc.2023.100726 PMC10721516

[85] Xu G, Ma H, Nei M, Kong H. Evolution of F-box genes in plants: different modes of sequence divergence and their relationships with functional diversification. Proc Natl Acad Sci U S A. 2009;106:835–40. 19126682 10.1073/pnas.0812043106 PMC2630105

[86] Annicchiarico P, Thami Alami I. Enhancing white lupin (*Lupinus albus* L.) adaptation to calcareous soils through lime-tolerant plant germplasm and Bradyrhizobium strains. Plant Soil. 2012;350:134–44.

[87] Annicchiarico P, Romani M, Barzaghi S, Ferrari B, Carroni AM, Ruda P, et al. Detection and exploitation of white lupin (*Lupinus albus* L.) genetic variation for gamma-conglutin. J Appl Bot Food Qual. 2016;89:212–6.

[88] Wink M, Hartmann T. Sites of enzymatic synthesis of quinolizidine alkaloids and their accumulation in *Lupinus polyphyllus*. Zeitschrift für Pflanzenphysiologie. 1981;102:337–44.

[89] Kirsten WJ, Hesselius GU. Rapid, automatic, high capacity Dumas determination of nitrogen. Microchem J. 1983;28:529–47.

[90] AOAC. Official Method of Analysis, Method 920.39, Fat (crude) or ether extract in animal feed. 18th edition. Gaithersburg, MD: AOAC International; 2005.

[91] Ferrari B, Barzaghi S, Annicchiarico P. Development of NIRS calibrations for seed content of lipids and proteins in contrasting white lupin germplasm. In: Chu X, Guo L, Huang Y, Yuan H, editors. Sense the Real Change: Proceedings of the 20th International Conference on Near Infrared. Singapore: Chemical Industry Press; 2022. pp. 132–6.

[92] Kennard RW, Stone LA. Computer aided design of experiments. Technometrics. 1969;11:137–48.

[93] Williams P. The RPD statistic: a tutorial note. NIR News. 2014;25:22–6.

[94] DeLacy IH, Basford KE, Cooper M, Bull IK, McLaren CG. Analysis of multi-environment trials – An historical perspective. In: Cooper M, Hammer GL, editors. Plant adaptation and crop improvement. Wallingford, UK: CABI; 1996. pp. 39–124.

[95] Itoh Y, Yamada Y. Relationships between genotype × environment interaction and genetic correlation of the same trait measured in different environments. Theor Appl Genet. 1990;80:11–6. 24220804 10.1007/BF00224009

[96] Robertson A. The sampling variance of the genetic correlation coefficient. Biometrics. 1959;15:469–85.

[97] SAS Institute. SAS/STAT 9.2 user’s guide. Cary, NC: SAS Institute; 2008.

[98] Hufnagel B, Marques A, Soriano A, Marquès L, Divol F, Doumas P, et al. High-quality genome sequence of white lupin provides insight into soil exploration and seed quality. Nat Commun. 2020;11:492. 31980615 10.1038/s41467-019-14197-9 PMC6981116

[99] Nazzicari N, Franguelli N, Ferrari B, Pecetti L, Annicchiarico P. The effect of genome parametrization and SNP marker subsetting on genomic selection in autotetraploid alfalfa. Genes. 2024;15:449. 38674384 10.3390/genes15040449 PMC11050091

[100] Nazzicari N, Biscarini F, Cozzi P, Brummer EC, Annicchiarico P. Marker imputation efficiency for genotyping-by-sequencing data in rice (*Oryza sativa*) and alfalfa (*Medicago sativa*). Mol Breed. 2016;36:69.

[101] Breiman L. Random forests. Mach Learn. 2001;45:5–32.

[102] Stekhoven DJ, Bühlmann P. Missforest–non-parametric missing value imputation for mixed-type data. Bioinformatics. 2012;28:112–8. 22039212 10.1093/bioinformatics/btr597

[103] Yendle PW, MacFie HJH. Discriminant principal components analysis. J Chemometrics. 1989;3:589–600.

[104] Jombart T, Ahmed. Adegenet 1.3-1: new tools for the analysis of genome-wide SNP data. Bioinformatics. 2011;27:3070–1. 21926124 10.1093/bioinformatics/btr521 PMC3198581

[105] Covarrubias-Pazaran G. Genome-assisted prediction of quantitative traits using the R package sommer. PLoS One. 2016;11:e0156744. 27271781 10.1371/journal.pone.0156744 PMC4894563

[106] Huang M, Liu X, Zhou Y, Summers RM, Zhang Z. BLINK: a package for the next level of genome-wide association studies with both individuals and markers in the millions. Gigascience. 2019;8:154. 10.1093/gigascience/giy154 PMC6365300 30535326

[107] Wang J, Zhang Z. GAPIT version 3: boosting power and accuracy for genomic association and prediction. Genomics Proteomics Bioinf. 2021;19:629–40. 10.1016/j.gpb.2021.08.005 PMC9121400 34492338

[108] Habier D, Fernando RL, Kizilkaya K, Garrick DJ. Extension of the bayesian alphabet for genomic selection. BMC Bioinformatics. 2011;12:1–12. 21605355 10.1186/1471-2105-12-186 PMC3144464

[109] Nazzicari N, Biscarini F. Stacked kinship CNN vs. GBLUP for genomic predictions of additive and complex continuous phenotypes. Sci Rep. 2022;12:19889. 36400808 10.1038/s41598-022-24405-0 PMC9674857

[110] Wang X, Xu Y, Hu Z, Xu C. Genomic selection methods for crop improvement: current status and prospects. Crop J. 2018;6:330–40.

