# Supplementary material for: Genetic variation and genome-enabled selection of white lupin for key seed quality traits
Source: BMC Genomics. 2025 Oct 15;26:922. doi: 10.1186/s12864-025-12048-0 (PMC12522229; doi:10.1186/s12864-025-12048-0)
Supplement: Supplementary file 1 — Supplementary Material 1. [file 12864_2025_12048_MOESM1_ESM.docx]

**Table S1** Average of best-linear unbiased prediction values of white lupin breeding lines for individual seed size and seed contents of protein, oil and total quinolizidine alkaloids (QA) recorded in different environments, for 16 factorial crosses between four landrace accession genotypes (LA parents) and four sweet-seed modern lines or cultivar (SL parents). Environment acronyms formed by the combination of LO = Lodi or TE = Temuco locations and cropping year (2018, 2019, 2020 or 2021)

|  |  | **Seed size (g)** | | |  | **Protein content (%)** | | | |  | **Oil content (%)** | | | |  | **QA (mg/kg)** |
| --- | --- | --- | --- | --- | --- | --- | --- | --- | --- | --- | --- | --- | --- | --- | --- | --- |
| **LA parent** | **SL parent** | **LO19** | **TE20** | **TE21** |  | **LO18** | **LO19** | **TE20** | **TE21** |  | **LO18** | **LO19** | **TE20** | **TE21** |  | **LO19** |
| GR56 | Lucky | 0.332 | 0.493 | 0.407 |  | 34.61 | 34.26 | 37.18 | 36.40 |  | 8.76 | 7.71 | 8.84 | 8.86 |  | 304.8 |
| GR56 | MB-38 | 0.325 | 0.461 | 0.412 |  | 35.84 | 35.27 | 37.79 | 36.63 |  | 9.29 | 8.70 | 9.06 | 8.57 |  | 472.1 |
| GR56 | Arsenio | 0.300 | 0.397 | 0.372 |  | 36.70 | 36.33 | 38.50 | 37.06 |  | 10.23 | 9.62 | 9.56 | 8.95 |  | 493.4 |
| GR56 | L27PS3 | 0.319 | 0.450 | 0.386 |  | 36.96 | 36.50 | 38.02 | 36.79 |  | 8.76 | 8.21 | 8.92 | 8.12 |  | 432.6 |
| La646 | Lucky | 0.386 | 0.483 | 0.393 |  | 36.49 | 35.97 | 37.89 | 36.56 |  | 8.40 | 7.23 | 8.64 | 8.13 |  | 290.0 |
| La646 | MB-38 | 0.382 | 0.459 | 0.381 |  | 36.48 | 35.73 | 37.69 | 36.86 |  | 9.34 | 8.65 | 9.75 | 9.21 |  | 468.0 |
| La646 | Arsenio | 0.323 | 0.402 | 0.358 |  | 36.14 | 36.75 | 38.17 | 36.78 |  | 9.74 | 8.76 | 9.13 | 8.30 |  | 423.4 |
| La646 | L27PS3 | 0.309 | 0.416 | 0.360 |  | 35.86 | 35.74 | 38.37 | 36.87 |  | 9.95 | 9.30 | 9.40 | 8.40 |  | 514.4 |
| La246 | Lucky | 0.406 | 0.620 | 0.471 |  | 36.72 | 36.09 | 37.93 | 37.00 |  | 9.08 | 8.39 | 9.24 | 9.02 |  | 342.4 |
| La246 | MB-38 | 0.313 | 0.395 | 0.350 |  | 33.37 | 34.95 | 38.17 | 36.82 |  | 9.57 | 8.48 | 10.00 | 9.28 |  | 244.1 |
| La246 | Arsenio | 0.366 | 0.461 | 0.397 |  | 35.14 | 35.81 | 38.24 | 37.03 |  | 10.39 | 9.77 | 9.71 | 9.03 |  | 400.9 |
| La246 | L27PS3 | 0.355 | 0.486 | 0.400 |  | 36.02 | 35.87 | 37.67 | 36.52 |  | 9.78 | 8.88 | 8.96 | 8.33 |  | 198.7 |
| La123 | Lucky | 0.451 | 0.599 | 0.463 |  | 36.30 | 36.50 | 37.33 | 36.46 |  | 8.86 | 7.91 | 8.23 | 8.27 |  | 131.4 |
| La123 | MB-38 | 0.418 | 0.535 | 0.409 |  | 36.47 | 36.29 | 37.33 | 36.28 |  | 9.29 | 8.87 | 8.05 | 7.56 |  | 406.0 |
| La123 | Arsenio | 0.450 | 0.545 | 0.447 |  | 35.79 | 35.78 | 37.45 | 36.24 |  | 9.74 | 9.13 | 9.41 | 8.79 |  | 232.5 |
| La123 | L27PS3 | 0.485 | 0.635 | 0.490 |  | 36.69 | 36.98 | 37.01 | 35.94 |  | 9.28 | 8.26 | 8.41 | 7.57 |  | 203.7 |
