# Supplementary material for: Genetic variation and genome-enabled selection of white lupin for key seed quality traits
Source: BMC Genomics. 2025 Oct 15;26:922. doi: 10.1186/s12864-025-12048-0 (PMC12522229; doi:10.1186/s12864-025-12048-0)
Supplement: Supplementary file 2 — Supplementary Material 2. [file 12864_2025_12048_MOESM2_ESM.docx]

**Table S3** List of white lupin genes potentially associated to significant SNPs detected by GWAS performed on landrace accessions or breeding lines (see Table 1 and Figs 2-4 for information on genotypes and evaluation environments). Candidate genes, identified by scanning a region up to the average chromosome distance at which linkage disequilibrium (*r^2^*) dropped to 0.1 in both directions from each SNP, are reported with their annotated function (https://www.whitelupin.fr/). The items highlighted in yellow are candidate genes having a role of potential interest in the alkaloid biosynthetic pathway according to literature, while those highlighted in green indicate SNPs close to the significance threshold but not significant (in case of both these occurrences, the SNP name is highlighted in green and the rest of the row in yellow)

| **Significant SNP** | **Trait** | **Dataset** | **Gene** | **Gene product** |
| --- | --- | --- | --- | --- |
| chr06_16367866 | seed weight | landraces | Lalb_Chr06g0175231 | putative protein |
| chr11_3050464 | seed weight | landraces | Lalb_Chr11g0066211 | Putative transcription factor C3H family |
| chr06_1599894 | protein | landraces | Lalb_Chr06g0163191 | Putative transcription factor C2H2 family |
| chr13_6963134 | seed weight | lines, Lodi | Lalb_Chr13g0293161 | hypothetical protein |
| chr13_6963134 | seed weight | lines, Lodi | Lalb_Chr13g0293171 | putative protein |
| chr10_17633340 | protein | lines, Temuco | Lalb_Chr10g0104741 | Putative S-adenosyl-L-methionine-dependent methyltransferase |
| chr10_17633340 | protein | lines, Temuco | Lalb_Chr10g0104751 | Putative S-adenosyl-L-methionine-dependent methyltransferase |
| chr09_6227890 | protein | lines, Temuco | Lalb_Chr09g0329981 | Putative acyl-CoA oxidase |
| chr07_5073784 | protein | lines, Temuco | Lalb_Chr07g0186561 | putative protein |
| chr03_1564676 | protein | lines, Lodi | Lalb_Chr03g0027211 | Putative dihydroorotase |
| chr03_1564676 | protein | lines, Lodi | Lalb_Chr03g0027221 | Putative FAS1 domain-containing protein |
| chr03_1564676 | protein | lines, Lodi | Lalb_Chr03g0027231 | Putative FAS1 domain-containing protein |
| chr03_1564676 | protein | lines, Lodi | Lalb_Chr03g0027241 | hypothetical protein |
| chr03_1564676 | protein | lines, Lodi | Lalb_Chr03g0027251 | Putative FAS1 domain-containing protein |
| chr13_15748617 | protein | lines, Lodi | Lalb_Chr13g0303931 | Putative DnaJ domain-containing protein |
| chr13_15748617 | protein | lines, Lodi | Lalb_Chr13g0303941 | tRNA-Pro |
| chr13_15748617 | protein | lines, Lodi | Lalb_Chr13g0303951 | Putative RNA recognition motif domain-containing protein |
| chr13_15748617 | protein | lines, Lodi | Lalb_Chr13g0303961 | Putative major facilitator, sugar transporter, major facilitator superfamily |
| chr02_2466588 | oil | lines, Temuco | Lalb_Chr02g0145211 | Putative TIP41-like protein |
| chr02_2466588 | oil | lines, Temuco | Lalb_Chr02g0145221 | Putative transcription factor C2H2 family |
| chr02_2466588 | oil | lines, Temuco | Lalb_Chr02g0145231 | hypothetical protein |
| chr07_5884176 | oil | lines, Lodi | Lalb_Chr07g0187601 | Putative transcription factor TCP family |
| chr07_5884176 | oil | lines, Lodi | Lalb_Chr07g0187611 | hypothetical protein |
| chr22_14984172 | oil | lines, Lodi | Lalb_Chr22g0361651 | Cycloartenol synthase |
| chr16_12303833 | oil | lines, Lodi | Lalb_Chr16g0390001 | Putative minus-end-directed kinesin ATPase |
| chr16_12303833 | oil | lines, Lodi | Lalb_Chr16g0390011 | Putative minus-end-directed kinesin ATPase |
| chr07_9145326 | oil | lines, Lodi | Lalb_Chr07g0190681 | Putative protein kinase CAMK-CAMKL-CHK1 family |
| chr01_18235431 | Total QA | lines, Lodi | Lalb_Chr01g0018101 | Probable small nuclear ribonucleoprotein G |
| chr01_18235431 | Total QA | lines, Lodi | Lalb_Chr01g0018111 | Putative F-box domain, kelch-type beta propeller |
| chr01_18235431 | Total QA | lines, Lodi | Lalb_Chr01g0018121 | Putative thioredoxin-like protein |
| chr23_480869 | albine | lines, Lodi | Lalb_Chr23g0265701 | Putative chromatin remodeling & transcriptional activation HMG family |
| chr23_480869 | albine | lines, Lodi | Lalb_Chr23g0265711 | Putative chromatin remodeler Bromodomain family |
| chr23_480869 | albine | lines, Lodi | Lalb_Chr23g0265721 | Putative Glycosyltransferase family 92, nucleotide-diphospho-sugar transferase |
| chr12_3447320 | albine | lines, Lodi | Lalb_Chr12g0201911 | Putative drought induced 19 type, zinc-binding protein |
| chr12_3447320 | albine | lines, Lodi | Lalb_Chr12g0201921 | putative protein |
| chr12_3447320 | albine | lines, Lodi | Lalb_Chr12g0201931 | Putative costunolide synthase |
| chr24_11264486 | albine | lines, Lodi | Lalb_Chr24g0402651 | Putative transcription factor MYB-HB-like family |
| chr09_3784402 | 13α-angeloyloxy lupanine | lines, Lodi | Lalb_Chr09g0326131 | putative protein |
| chr09_3784402 | 13α-angeloyloxy lupanine | lines, Lodi | Lalb_Chr09g0326141 | Putative 43kDa postsynaptic protein |
| chr21_1801842 | 13α-angeloyloxy lupanine | lines, Lodi | Lalb_Chr21g0307581 | Putative non-specific protein-tyrosine kinase RLK-Pelle-RLCK-VIII family |
| chr21_1801842 | 13α-angeloyloxy lupanine | lines, Lodi | Lalb_Chr21g0307591 | tRNA-Ala |
| chr21_1801842 | 13α-angeloyloxy lupanine | lines, Lodi | Lalb_Chr21g0307601 | survival of motor neuron protein-interacting protein 1 (GEMIN2) |
| chr21_1801842 | 13α-angeloyloxy lupanine | lines, Lodi | Lalb_Chr21g0307611 | Putative nucleotide-sugar transporter |
| chr11_873806 | 13α-angeloyloxy lupanine | lines, Lodi | Lalb_Chr11g0062801 | putative protein |
| chr11_873806 | 13α-angeloyloxy lupanine | lines, Lodi | Lalb_Chr11g0062811 | Putative oxidoreductase |
| chr21_2683307 | angustifoline | lines, Lodi | Lalb_Chr21g0308971 | hypothetical protein |
| chr21_2683307 | angustifoline | lines, Lodi | Lalb_Chr21g0308981 | Putative UV excision repair protein Rad23 |
| chr21_2683307 | angustifoline | lines, Lodi | Lalb_Chr21g0308991 | Putative glycolipid transfer protein |
| chr23_7831318 | angustifoline | lines, Lodi | Lalb_Chr23g0274131 | Putative RNA helicase |
| chr07_2889068 | angustifoline | lines, Lodi | Lalb_Chr07g0182521 | betaine-aldehyde dehydrogenase (betB, gbsA) |
| chr07_2889068 | angustifoline | lines, Lodi | Lalb_Chr07g0182531 | Putative oxidoreductase |
| chr21_1801842 | angustifoline | lines, Lodi | Lalb_Chr21g0307581 | Putative non-specific protein-tyrosine kinase RLK-Pelle-RLCK-VIII family |
| chr21_1801842 | angustifoline | lines, Lodi | Lalb_Chr21g0307591 | tRNA-Ala |
| chr21_1801842 | angustifoline | lines, Lodi | Lalb_Chr21g0307601 | survival of motor neuron protein-interacting protein 1 (GEMIN2) |
| chr21_1801842 | angustifoline | lines, Lodi | Lalb_Chr21g0307611 | Putative nucleotide-sugar transporter |
| chr21_2683307 | 13α-OH lupanine | lines, Lodi | Lalb_Chr21g0308971 | hypothetical protein |
| chr21_2683307 | 13α-OH lupanine | lines, Lodi | Lalb_Chr21g0308981 | Putative UV excision repair protein Rad23 |
| chr21_2683307 | 13α-OH lupanine | lines, Lodi | Lalb_Chr21g0308991 | Putative glycolipid transfer protein |
| chr07_3539326 | 13α-OH lupanine | lines, Lodi | Lalb_Chr07g0183781 | Putative signal recognition particle subunit SRP68 |
| chr07_3539326 | 13α-OH lupanine | lines, Lodi | Lalb_Chr07g0183791 | hypothetical protein |
| chr07_3539326 | 13α-OH lupanine | lines, Lodi | Lalb_Chr07g0183801 | Putative transcription factor MYB-HB-like family |
| chr03_18819666 | 13α-OH lupanine | lines, Lodi | Lalb_Chr03g0041081 | Putative gamma-tubulin complex component protein |
| chr05_6178718 | 13α-OH multiflorine | lines, Lodi | Lalb_Chr05g0221611 | Putative phosphate-transporting ATPase |
| chr05_6178718 | 13α-OH multiflorine | lines, Lodi | Lalb_Chr05g0221621 | SEL1 protein/HCP-like superfamily protein-related |
| chr12_3542613 | lupanine | lines, Lodi | Lalb_Chr12g0202051 | Putative initiation factor eIF-4 gamma, MA3 |
| chr12_3542613 | lupanine | lines, Lodi | Lalb_Chr12g0202061 | Putative nucleotide exchange factor Fes1 |
| chr12_3542613 | lupanine | lines, Lodi | Lalb_Chr12g0202071 | Putative [Histone H3]-lysine-36 demethylase |
| chr03_3330272 | lupanine | lines, Lodi | Lalb_Chr03g0030441 | Putative 1,4-dihydroxy-2-naphthoyl-CoA hydrolase |
| chr03_3330272 | lupanine | lines, Lodi | Lalb_Chr03g0030451 | Root-cap (LEA protein) |
| chr03_3330272 | lupanine | lines, Lodi | Lalb_Chr03g0030461 | Putative START-like domain, Bet v I type allergen |
| chr14_10375114 | multiflorine | lines, Lodi | Lalb_Chr14g0368801 | Putative succinate dehydrogenase (quinone) |
| chr14_10375114 | multiflorine | lines, Lodi | Lalb_Chr14g0368811 | Putative carboxylesterase |
| chr14_10375114 | multiflorine | lines, Lodi | Lalb_Chr14g0368821 | Putative carboxylesterase |
| chr14_10375114 | multiflorine | lines, Lodi | Lalb_Chr14g0368831 | Putative P-loop containing nucleoside triphosphate hydrolase |
| chr10_15854177 | multiflorine | lines, Lodi | Lalb_Chr10g0101471 | Putative histone-lysine N-methyltransferase chromatin regulator PHD family |
| chr10_15854177 | multiflorine | lines, Lodi | Lalb_Chr10g0101481 | Putative S-adenosyl-L-methionine-dependent methyltransferase |
| chr21_5455243 | multiflorine | lines, Lodi | Lalb_Chr21g0313201 | Putative glucomannan 4-beta-mannosyltransferase |
| chr15_18089590 | multiflorine | lines, Lodi | Lalb_Chr15g0087401 | Putative 3-dehydrosphinganine reductase |
| chr03_20606439 | multiflorine | lines, Lodi | Lalb_Chr03g0043001 | Putative pentatricopeptide |
| chr03_20606439 | multiflorine | lines, Lodi | Lalb_Chr03g0043011 | hypothetical protein |
| chr03_20606439 | multiflorine | lines, Lodi | Lalb_Chr03g0043021 | Putative peptide-transporting ATPase |
| chr03_20606439 | multiflorine | lines, Lodi | Lalb_Chr03g0043031 | Putative transcription factor MYB/SANT family |
| chr10_14376008 | N-methyl albine | lines, Lodi | Lalb_Chr10g0099251 | Putative 1-phosphatidylinositol 4-kinase |
| chr20_5975694 | N-methyl albine | lines, Lodi | Lalb_Chr20g0114681 | Putative S-adenosyl-L-methionine-dependent methyltransferase |
| chr25_9303017 | oxolupanine | lines, Lodi | Lalb_Chr25g0282621 | Putative Seed maturation protein |
| chr25_9303017 | oxolupanine | lines, Lodi | Lalb_Chr25g0282631 | Putative Seed maturation protein |
| chr09_3961562 | oxolupanine | lines, Lodi | Lalb_Chr09g0326431 | Putative adaptor protein complex AP-1, gamma subunit |
| chr18_2394041 | oxolupanine | lines, Lodi | Lalb_Chr18g0047311 | Putative transcription factor C3H family |
| chr18_2394041 | oxolupanine | lines, Lodi | Lalb_Chr18g0047321 | Putative SKP1/BTB/POZ domain, NPH3 domain-containing protein |
| chr18_2394041 | oxolupanine | lines, Lodi | Lalb_Chr18g0047331 | Putative thioredoxin-disulfide reductase |
| chr18_2394041 | oxolupanine | lines, Lodi | Lalb_Chr18g0047341 | Putative tRNA pseudouridine(38-40) synthase |
| chr01_2263350 | oxolupanine | lines, Lodi | Lalb_Chr01g0004321 | tRNA-Lys |
| chr01_2263350 | oxolupanine | lines, Lodi | Lalb_Chr01g0004331 | Putative SIT4 phosphatase-associated protein family |
| chr21_2683307 | 13α-tigloyloxy lupanine | lines, Lodi | Lalb_Chr21g0308971 | hypothetical protein |
| chr21_2683307 | 13α-tigloyloxy lupanine | lines, Lodi | Lalb_Chr21g0308981 | Putative UV excision repair protein Rad23 |
| chr21_2683307 | 13α-tigloyloxy lupanine | lines, Lodi | Lalb_Chr21g0308991 | Putative glycolipid transfer protein |
| chr03_2039883 | 13α-tigloyloxy lupanine | lines, Lodi | Lalb_Chr03g0028121 | Putative von Willebrand factor, type A, Zinc finger, Sec23/Sec24-type, sec23/Sec24, trunk |
| chr03_2039883 | 13α-tigloyloxy lupanine | lines, Lodi | Lalb_Chr03g0028131 | Putative Rho GTPase activation protein |
