# Supplementary material for: Genetic variation and genome-enabled selection of white lupin for key seed quality traits
Source: BMC Genomics. 2025 Oct 15;26:922. doi: 10.1186/s12864-025-12048-0 (PMC12522229; doi:10.1186/s12864-025-12048-0)
Supplement: Supplementary file 3 — Supplementary Material 3. [file 12864_2025_12048_MOESM3_ESM.docx]

**Table S2** Phenotypic correlation coefficients for total content of quinolizidine alkaloids (QAs) and individual QAs of 142 white lupin breeding lines evaluated in Lodi (Italy) (based on best-linear unbiased prediction values)

| QA | Code | Total QAs | A1 | A2 | A3 | A4 | A5 | A6 | A7 | A8 | A9 | A10 | A11 | A12 |
| --- | --- | --- | --- | --- | --- | --- | --- | --- | --- | --- | --- | --- | --- | --- |
| Lupanine | A1 | 0.92 | − | − | − | − | − | − | − | − | − | − | − | − |
| 13α-hydroxylupanine | A2 | 0.68 | 0.40 | − | − | − | − | − | − | − | − | − | − | − |
| 13α-angeloyloxylupanine | A3 | 0.56 | 0.25 | 0.76 | − | − | − | − | − | − | − | − | − | − |
| Angustifoline | A4 | 0.66 | 0.38 | 0.91 | 0.80 | − | − | − | − | − | − | − | − | − |
| N-methylalbine | A5 | 0.40 | 0.29 | 0.23 | 0.13 | 0.17 | − | − | − | − | − | − | − | − |
| α-isolupanine | A6 | 0.87 | 0.67 | 0.75 | 0.70 | 0.73 | 0.51 | − | − | − | − | − | − | − |
| Ammodendrine | A7 | 0.61 | 0.51 | 0.37 | 0.31 | 0.41 | 0.50 | 0.59 | − | − | − | − | − | − |
| 13α-tigloyloxylupanine | A8 | 0.58 | 0.29 | 0.78 | 0.79 | 0.78 | 0.28 | 0.69 | 0.44 | − | − | − | − | − |
| Multiflorine | A9 | 0.27 | 0.14 | 0.15 | 0.30 | 0.18 | 0.34 | 0.39 | 0.38 | 0.22 | − | − | − | − |
| Tetrahydrorhombifoline | A10 | 0.54 | 0.28 | 0.62 | 0.64 | 0.61 | 0.49 | 0.72 | 0.50 | 0.67 | 0.36 | − | − | − |
| 17-oxolupanine | A11 | 0.56 | 0.46 | 0.46 | 0.44 | 0.44 | 0.18 | 0.49 | 0.33 | 0.35 | 0.17 | 0.45 | − | − |
| 13-hydroxymultiflorine | A12 | 0.41 | 0.22 | 0.40 | 0.60 | 0.48 | 0.32 | 0.53 | 0.35 | 0.51 | 0.35 | 0.53 | 0.40 | − |
| Albine | A13 | 0.48 | 0.43 | 0.23 | 0.09 | 0.19 | 0.79 | 0.47 | 0.45 | 0.18 | 0.25 | 0.35 | 0.23 | 0.22 |

Values ≥ 0.17 are significant at *P* > 0.05; values ≥ 0.22 are significant at P < 0.01.
