# Supplementary figures and images for: Genetic variation and genome-enabled selection of white lupin for key seed quality traits
Source: BMC Genomics. 2025 Oct 15;26:922. doi: 10.1186/s12864-025-12048-0 (PMC12522229; doi:10.1186/s12864-025-12048-0)

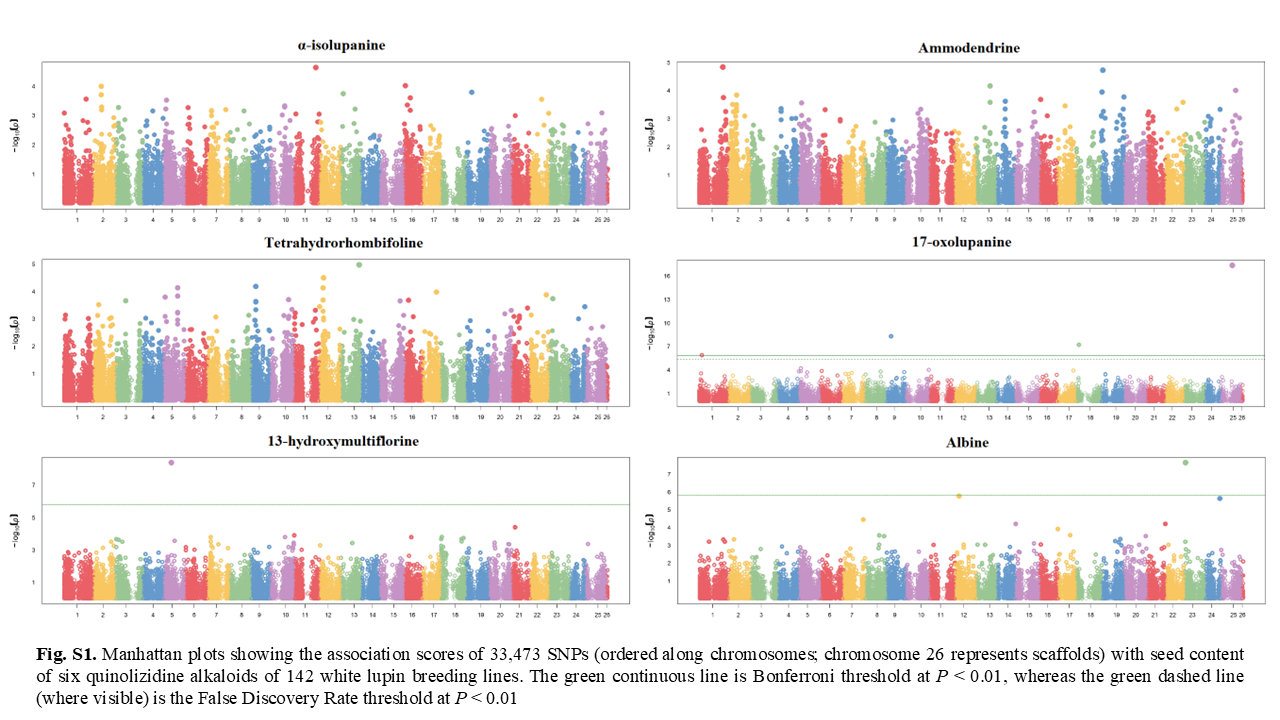

Supplement: Supplementary file 4 — Supplementary Material 4. [file 12864_2025_12048_MOESM4_ESM.tif]

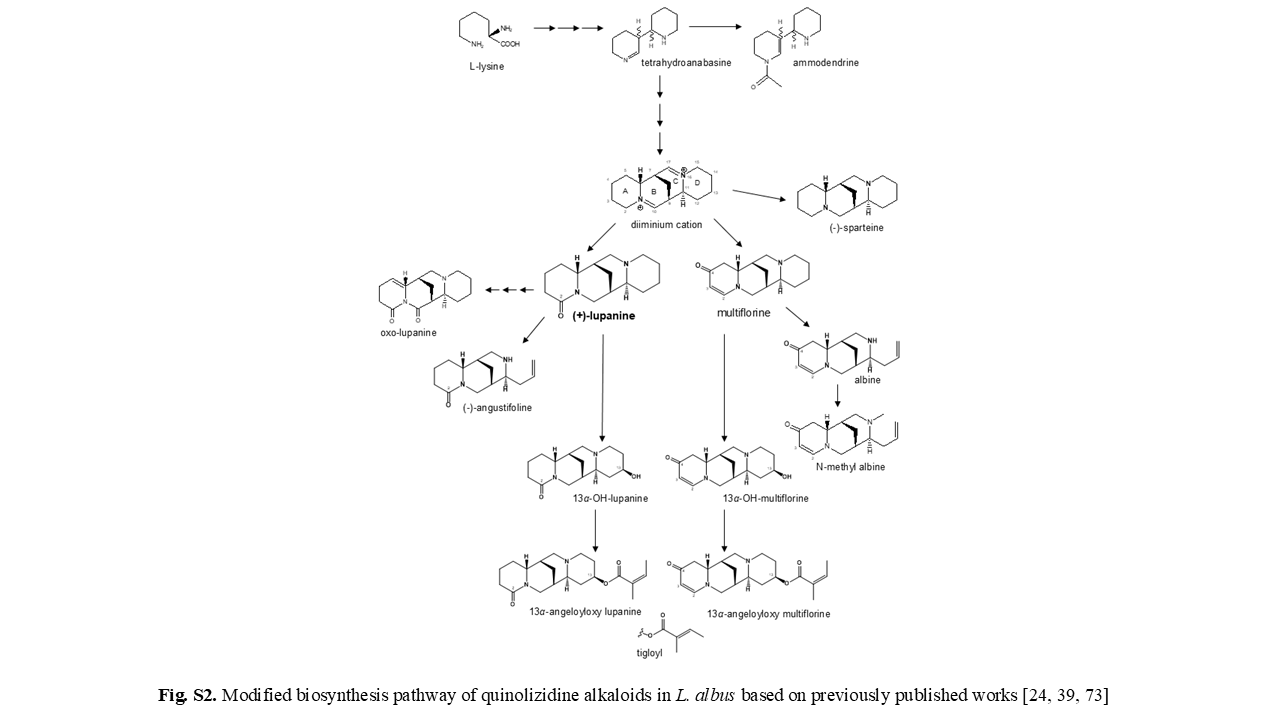

Supplement: Supplementary file 5 — Supplementary Material 5. [file 12864_2025_12048_MOESM5_ESM.tif]

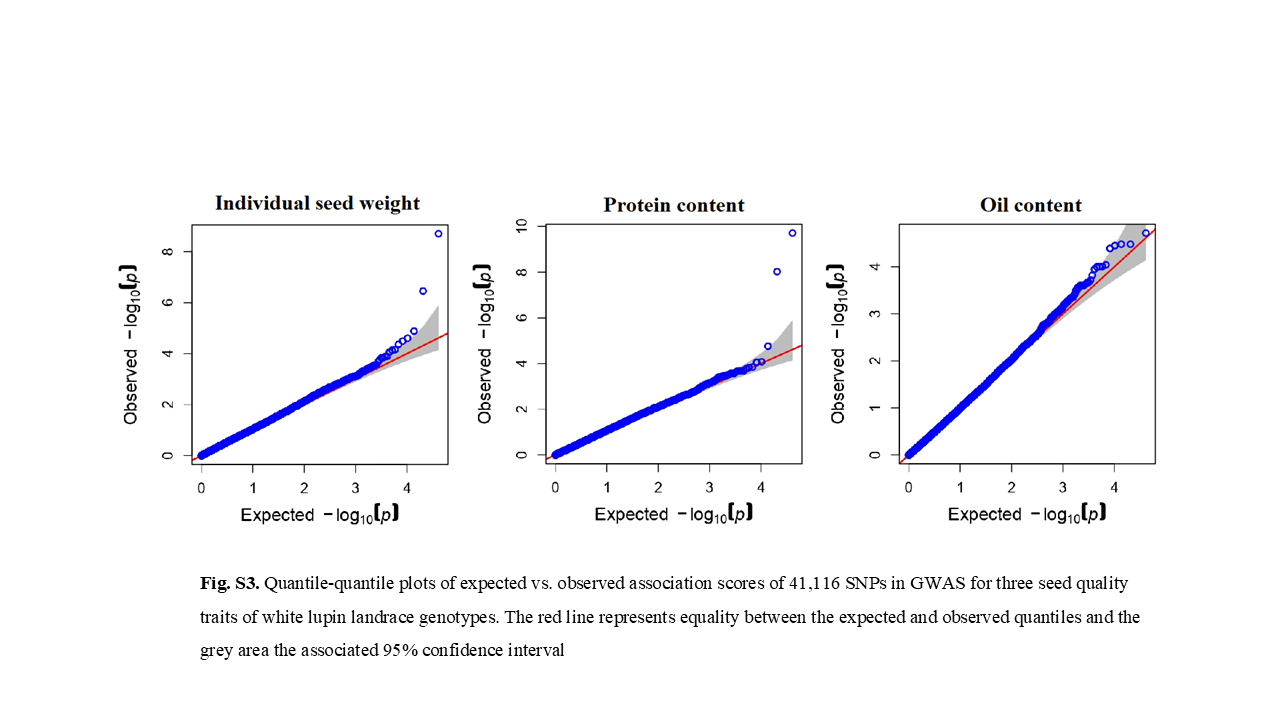

Supplement: Supplementary file 6 — Supplementary Material 6. [file 12864_2025_12048_MOESM6_ESM.tif]

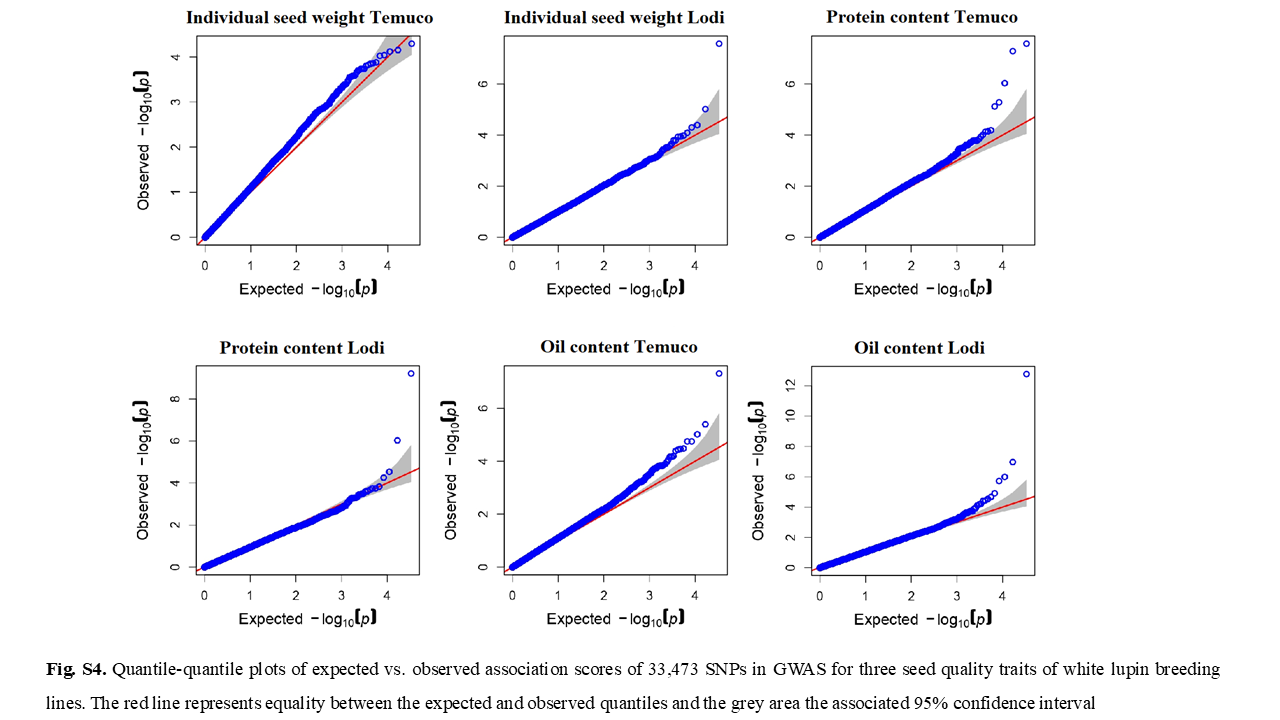

Supplement: Supplementary file 7 — Supplementary Material 7. [file 12864_2025_12048_MOESM7_ESM.tif]

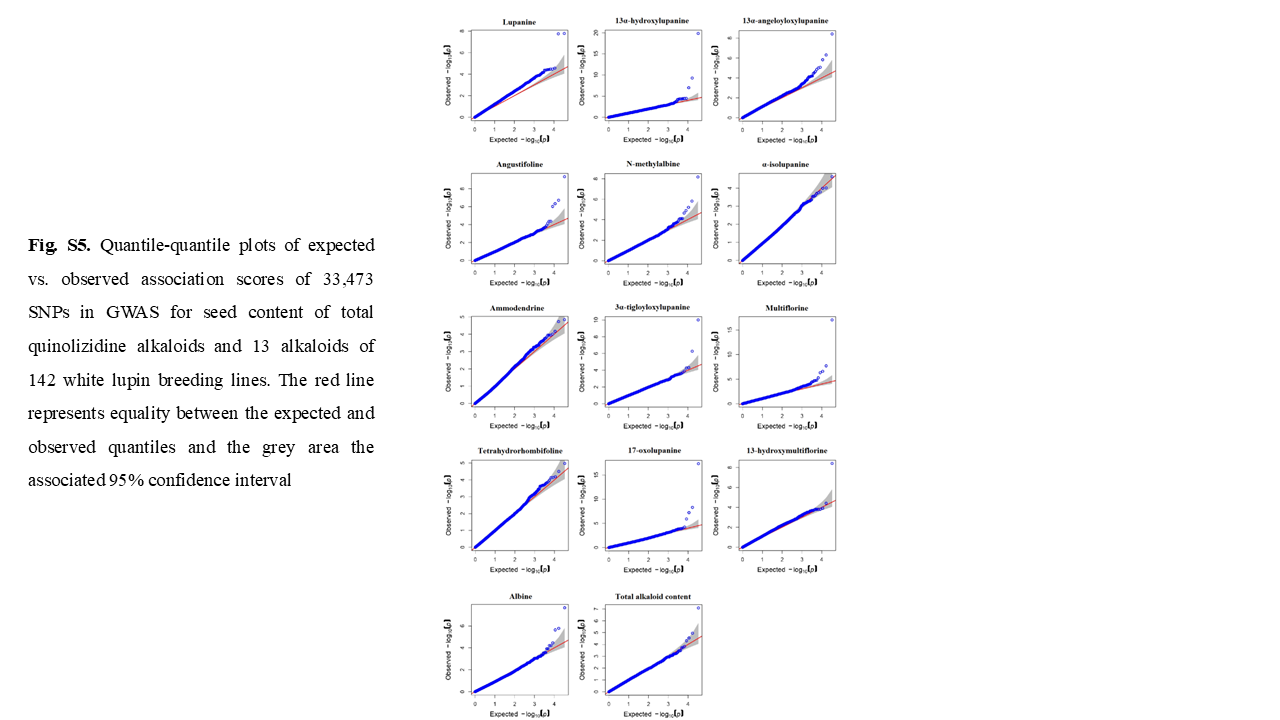

Supplement: Supplementary file 8 — Supplementary Material 8. [file 12864_2025_12048_MOESM8_ESM.tif]
